# Search for doubly charged Higgs boson production in multi-lepton final states with the ATLAS detector using proton–proton collisions at $\sqrt{s}=13\,\text {TeV}$

**DOI:** 10.1140/epjc/s10052-018-5661-z

**Published:** 2018-03-10

**Authors:** M. Aaboud, G. Aad, B. Abbott, O. Abdinov, B. Abeloos, S. H. Abidi, O. S. AbouZeid, N. L. Abraham, H. Abramowicz, H. Abreu, R. Abreu, Y. Abulaiti, B. S. Acharya, S. Adachi, L. Adamczyk, J. Adelman, M. Adersberger, T. Adye, A. A. Affolder, Y. Afik, T. Agatonovic-Jovin, C. Agheorghiesei, J. A. Aguilar-Saavedra, S. P. Ahlen, F. Ahmadov, G. Aielli, S. Akatsuka, H. Akerstedt, T. P. A. Åkesson, E. Akilli, A. V. Akimov, G. L. Alberghi, J. Albert, P. Albicocco, M. J. Alconada Verzini, S. C. Alderweireldt, M. Aleksa, I. N. Aleksandrov, C. Alexa, G. Alexander, T. Alexopoulos, M. Alhroob, B. Ali, M. Aliev, G. Alimonti, J. Alison, S. P. Alkire, B. M. M. Allbrooke, B. W. Allen, P. P. Allport, A. Aloisio, A. Alonso, F. Alonso, C. Alpigiani, A. A. Alshehri, M. I. Alstaty, B. Alvarez Gonzalez, D. Álvarez Piqueras, M. G. Alviggi, B. T. Amadio, Y. Amaral Coutinho, C. Amelung, D. Amidei, S. P. Amor Dos Santos, S. Amoroso, G. Amundsen, C. Anastopoulos, L. S. Ancu, N. Andari, T. Andeen, C. F. Anders, J. K. Anders, K. J. Anderson, A. Andreazza, V. Andrei, S. Angelidakis, I. Angelozzi, A. Angerami, A. V. Anisenkov, N. Anjos, A. Annovi, C. Antel, M. Antonelli, A. Antonov, D. J. Antrim, F. Anulli, M. Aoki, L. Aperio Bella, G. Arabidze, Y. Arai, J. P. Araque, V. Araujo Ferraz, A. T. H. Arce, R. E. Ardell, F. A. Arduh, J-F. Arguin, S. Argyropoulos, M. Arik, A. J. Armbruster, L. J. Armitage, O. Arnaez, H. Arnold, M. Arratia, O. Arslan, A. Artamonov, G. Artoni, S. Artz, S. Asai, N. Asbah, A. Ashkenazi, L. Asquith, K. Assamagan, R. Astalos, M. Atkinson, N. B. Atlay, K. Augsten, G. Avolio, B. Axen, M. K. Ayoub, G. Azuelos, A. E. Baas, M. J. Baca, H. Bachacou, K. Bachas, M. Backes, P. Bagnaia, M. Bahmani, H. Bahrasemani, J. T. Baines, M. Bajic, O. K. Baker, P. J. Bakker, E. M. Baldin, P. Balek, F. Balli, W. K. Balunas, E. Banas, A. Bandyopadhyay, Sw. Banerjee, A. A. E. Bannoura, L. Barak, E. L. Barberio, D. Barberis, M. Barbero, T. Barillari, M-S. Barisits, J. T. Barkeloo, T. Barklow, N. Barlow, S. L. Barnes, B. M. Barnett, R. M. Barnett, Z. Barnovska-Blenessy, A. Baroncelli, G. Barone, A. J. Barr, L. Barranco Navarro, F. Barreiro, J. Barreiro Guimarães da Costa, R. Bartoldus, A. E. Barton, P. Bartos, A. Basalaev, A. Bassalat, R. L. Bates, S. J. Batista, J. R. Batley, M. Battaglia, M. Bauce, F. Bauer, H. S. Bawa, J. B. Beacham, M. D. Beattie, T. Beau, P. H. Beauchemin, P. Bechtle, H. P. Beck, H. C. Beck, K. Becker, M. Becker, C. Becot, A. J. Beddall, A. Beddall, V. A. Bednyakov, M. Bedognetti, C. P. Bee, T. A. Beermann, M. Begalli, M. Begel, J. K. Behr, A. S. Bell, G. Bella, L. Bellagamba, A. Bellerive, M. Bellomo, K. Belotskiy, O. Beltramello, N. L. Belyaev, O. Benary, D. Benchekroun, M. Bender, N. Benekos, Y. Benhammou, E. Benhar Noccioli, J. Benitez, D. P. Benjamin, M. Benoit, J. R. Bensinger, S. Bentvelsen, L. Beresford, M. Beretta, D. Berge, E. Bergeaas Kuutmann, N. Berger, L. J. Bergsten, J. Beringer, S. Berlendis, N. R. Bernard, G. Bernardi, C. Bernius, F. U. Bernlochner, T. Berry, P. Berta, C. Bertella, G. Bertoli, I. A. Bertram, C. Bertsche, G. J. Besjes, O. Bessidskaia Bylund, M. Bessner, N. Besson, A. Bethani, S. Bethke, A. Betti, A. J. Bevan, J. Beyer, R. M. Bianchi, O. Biebel, D. Biedermann, R. Bielski, K. Bierwagen, N. V. Biesuz, M. Biglietti, T. R. V. Billoud, H. Bilokon, M. Bindi, A. Bingul, C. Bini, S. Biondi, T. Bisanz, C. Bittrich, D. M. Bjergaard, J. E. Black, K. M. Black, R. E. Blair, T. Blazek, I. Bloch, C. Blocker, A. Blue, U. Blumenschein, S. Blunier, G. J. Bobbink, V. S. Bobrovnikov, S. S. Bocchetta, A. Bocci, C. Bock, M. Boehler, D. Boerner, D. Bogavac, A. G. Bogdanchikov, C. Bohm, V. Boisvert, P. Bokan, T. Bold, A. S. Boldyrev, A. E. Bolz, M. Bomben, M. Bona, M. Boonekamp, A. Borisov, G. Borissov, J. Bortfeldt, D. Bortoletto, V. Bortolotto, D. Boscherini, M. Bosman, J. D. Bossio Sola, J. Boudreau, E. V. Bouhova-Thacker, D. Boumediene, C. Bourdarios, S. K. Boutle, A. Boveia, J. Boyd, I. R. Boyko, A. J. Bozson, J. Bracinik, A. Brandt, G. Brandt, O. Brandt, F. Braren, U. Bratzler, B. Brau, J. E. Brau, W. D. Breaden Madden, K. Brendlinger, A. J. Brennan, L. Brenner, R. Brenner, S. Bressler, D. L. Briglin, T. M. Bristow, D. Britton, D. Britzger, F. M. Brochu, I. Brock, R. Brock, G. Brooijmans, T. Brooks, W. K. Brooks, J. Brosamer, E. Brost, J. H Broughton, P. A. Bruckman de Renstrom, D. Bruncko, A. Bruni, G. Bruni, L. S. Bruni, S. Bruno, BH Brunt, M. Bruschi, N. Bruscino, P. Bryant, L. Bryngemark, T. Buanes, Q. Buat, P. Buchholz, A. G. Buckley, I. A. Budagov, F. Buehrer, M. K. Bugge, O. Bulekov, D. Bullock, T. J. Burch, S. Burdin, C. D. Burgard, A. M. Burger, B. Burghgrave, K. Burka, S. Burke, I. Burmeister, J. T. P. Burr, D. Büscher, V. Büscher, P. Bussey, J. M. Butler, C. M. Buttar, J. M. Butterworth, P. Butti, W. Buttinger, A. Buzatu, A. R. Buzykaev, S. Cabrera Urbán, D. Caforio, H. Cai, V. M. Cairo, O. Cakir, N. Calace, P. Calafiura, A. Calandri, G. Calderini, P. Calfayan, G. Callea, L. P. Caloba, S. Calvente Lopez, D. Calvet, S. Calvet, T. P. Calvet, R. Camacho Toro, S. Camarda, P. Camarri, D. Cameron, R. Caminal Armadans, C. Camincher, S. Campana, M. Campanelli, A. Camplani, A. Campoverde, V. Canale, M. Cano Bret, J. Cantero, T. Cao, M. D. M. Capeans Garrido, I. Caprini, M. Caprini, M. Capua, R. M. Carbone, R. Cardarelli, F. Cardillo, I. Carli, T. Carli, G. Carlino, B. T. Carlson, L. Carminati, R. M. D. Carney, S. Caron, E. Carquin, S. Carrá, G. D. Carrillo-Montoya, D. Casadei, M. P. Casado, A. F. Casha, M. Casolino, D. W. Casper, R. Castelijn, V. Castillo Gimenez, N. F. Castro, A. Catinaccio, J. R. Catmore, A. Cattai, J. Caudron, V. Cavaliere, E. Cavallaro, D. Cavalli, M. Cavalli-Sforza, V. Cavasinni, E. Celebi, F. Ceradini, L. Cerda Alberich, A. S. Cerqueira, A. Cerri, L. Cerrito, F. Cerutti, A. Cervelli, S. A. Cetin, A. Chafaq, D. Chakraborty, S. K. Chan, W. S. Chan, Y. L. Chan, P. Chang, J. D. Chapman, D. G. Charlton, C. C. Chau, C. A. Chavez Barajas, S. Che, S. Cheatham, A. Chegwidden, S. Chekanov, S. V. Chekulaev, G. A. Chelkov, M. A. Chelstowska, C. Chen, C. Chen, H. Chen, J. Chen, S. Chen, S. Chen, X. Chen, Y. Chen, H. C. Cheng, H. J. Cheng, A. Cheplakov, E. Cheremushkina, R. Cherkaoui El Moursli, E. Cheu, K. Cheung, L. Chevalier, V. Chiarella, G. Chiarelli, G. Chiodini, A. S. Chisholm, A. Chitan, Y. H. Chiu, M. V. Chizhov, K. Choi, A. R. Chomont, S. Chouridou, Y. S. Chow, V. Christodoulou, M. C. Chu, J. Chudoba, A. J. Chuinard, J. J. Chwastowski, L. Chytka, A. K. Ciftci, D. Cinca, V. Cindro, I. A. Cioara, A. Ciocio, F. Cirotto, Z. H. Citron, M. Citterio, M. Ciubancan, A. Clark, B. L. Clark, M. R. Clark, P. J. Clark, R. N. Clarke, C. Clement, Y. Coadou, M. Cobal, A. Coccaro, J. Cochran, L. Colasurdo, B. Cole, A. P. Colijn, J. Collot, T. Colombo, P. Conde Muiño, E. Coniavitis, S. H. Connell, I. A. Connelly, S. Constantinescu, G. Conti, F. Conventi, M. Cooke, A. M. Cooper-Sarkar, F. Cormier, K. J. R. Cormier, M. Corradi, F. Corriveau, A. Cortes-Gonzalez, G. Costa, M. J. Costa, D. Costanzo, G. Cottin, G. Cowan, B. E. Cox, K. Cranmer, S. J. Crawley, R. A. Creager, G. Cree, S. Crépé-Renaudin, F. Crescioli, W. A. Cribbs, M. Cristinziani, V. Croft, G. Crosetti, A. Cueto, T. Cuhadar Donszelmann, A. R. Cukierman, J. Cummings, M. Curatolo, J. Cúth, S. Czekierda, P. Czodrowski, G. D’amen, S. D’Auria, L. D’eramo, M. D’Onofrio, M. J. Da Cunha Sargedas De Sousa, C. Da Via, W. Dabrowski, T. Dado, T. Dai, O. Dale, F. Dallaire, C. Dallapiccola, M. Dam, J. R. Dandoy, M. F. Daneri, N. P. Dang, A. C. Daniells, N. S. Dann, M. Danninger, M. Dano Hoffmann, V. Dao, G. Darbo, S. Darmora, J. Dassoulas, A. Dattagupta, T. Daubney, W. Davey, C. David, T. Davidek, D. R. Davis, P. Davison, E. Dawe, I. Dawson, K. De, R. de Asmundis, A. De Benedetti, S. De Castro, S. De Cecco, N. De Groot, P. de Jong, H. De la Torre, F. De Lorenzi, A. De Maria, D. De Pedis, A. De Salvo, U. De Sanctis, A. De Santo, K. De Vasconcelos Corga, J. B. De Vivie De Regie, R. Debbe, C. Debenedetti, D. V. Dedovich, N. Dehghanian, I. Deigaard, M. Del Gaudio, J. Del Peso, D. Delgove, F. Deliot, C. M. Delitzsch, A. Dell’Acqua, L. Dell’Asta, M. Dell’Orso, M. Della Pietra, D. della Volpe, M. Delmastro, C. Delporte, P. A. Delsart, D. A. DeMarco, S. Demers, M. Demichev, A. Demilly, S. P. Denisov, D. Denysiuk, D. Derendarz, J. E. Derkaoui, F. Derue, P. Dervan, K. Desch, C. Deterre, K. Dette, M. R. Devesa, P. O. Deviveiros, A. Dewhurst, S. Dhaliwal, F. A. Di Bello, A. Di Ciaccio, L. Di Ciaccio, W. K. Di Clemente, C. Di Donato, A. Di Girolamo, B. Di Girolamo, B. Di Micco, R. Di Nardo, K. F. Di Petrillo, A. Di Simone, R. Di Sipio, D. Di Valentino, C. Diaconu, M. Diamond, F. A. Dias, M. A. Diaz, E. B. Diehl, J. Dietrich, S. Díez Cornell, A. Dimitrievska, J. Dingfelder, P. Dita, S. Dita, F. Dittus, F. Djama, T. Djobava, J. I. Djuvsland, M. A. B. do Vale, D. Dobos, M. Dobre, D. Dodsworth, C. Doglioni, J. Dolejsi, Z. Dolezal, M. Donadelli, S. Donati, P. Dondero, J. Donini, J. Dopke, A. Doria, M. T. Dova, A. T. Doyle, E. Drechsler, M. Dris, Y. Du, J. Duarte-Campderros, F. Dubinin, A. Dubreuil, E. Duchovni, G. Duckeck, A. Ducourthial, O. A. Ducu, D. Duda, A. Dudarev, A. Chr. Dudder, E. M. Duffield, L. Duflot, M. Dührssen, C. Dulsen, M. Dumancic, A. E. Dumitriu, A. K. Duncan, M. Dunford, A. Duperrin, H. Duran Yildiz, M. Düren, A. Durglishvili, D. Duschinger, B. Dutta, D. Duvnjak, M. Dyndal, B. S. Dziedzic, C. Eckardt, K. M. Ecker, R. C. Edgar, T. Eifert, G. Eigen, K. Einsweiler, T. Ekelof, M. El Kacimi, R. El Kosseifi, V. Ellajosyula, M. Ellert, S. Elles, F. Ellinghaus, A. A. Elliot, N. Ellis, J. Elmsheuser, M. Elsing, D. Emeliyanov, Y. Enari, J. S. Ennis, M. B. Epland, J. Erdmann, A. Ereditato, M. Ernst, S. Errede, M. Escalier, C. Escobar, B. Esposito, O. Estrada Pastor, A. I. Etienvre, E. Etzion, H. Evans, A. Ezhilov, M. Ezzi, F. Fabbri, L. Fabbri, V. Fabiani, G. Facini, R. M. Fakhrutdinov, S. Falciano, R. J. Falla, J. Faltova, Y. Fang, M. Fanti, A. Farbin, A. Farilla, C. Farina, E. M. Farina, T. Farooque, S. Farrell, S. M. Farrington, P. Farthouat, F. Fassi, P. Fassnacht, D. Fassouliotis, M. Faucci Giannelli, A. Favareto, W. J. Fawcett, L. Fayard, O. L. Fedin, W. Fedorko, S. Feigl, L. Feligioni, C. Feng, E. J. Feng, M. J. Fenton, A. B. Fenyuk, L. Feremenga, P. Fernandez Martinez, J. Ferrando, A. Ferrari, P. Ferrari, R. Ferrari, D. E. Ferreira de Lima, A. Ferrer, D. Ferrere, C. Ferretti, F. Fiedler, A. Filipčič, M. Filipuzzi, F. Filthaut, M. Fincke-Keeler, K. D. Finelli, M. C. N. Fiolhais, L. Fiorini, A. Fischer, C. Fischer, J. Fischer, W. C. Fisher, N. Flaschel, I. Fleck, P. Fleischmann, R. R. M. Fletcher, T. Flick, B. M. Flierl, L. R. Flores Castillo, M. J. Flowerdew, G. T. Forcolin, A. Formica, F. A. Förster, A. Forti, A. G. Foster, D. Fournier, H. Fox, S. Fracchia, P. Francavilla, M. Franchini, S. Franchino, D. Francis, L. Franconi, M. Franklin, M. Frate, M. Fraternali, D. Freeborn, S. M. Fressard-Batraneanu, B. Freund, D. Froidevaux, J. A. Frost, C. Fukunaga, T. Fusayasu, J. Fuster, O. Gabizon, A. Gabrielli, A. Gabrielli, G. P. Gach, S. Gadatsch, S. Gadomski, G. Gagliardi, L. G. Gagnon, C. Galea, B. Galhardo, E. J. Gallas, B. J. Gallop, P. Gallus, G. Galster, K. K. Gan, S. Ganguly, Y. Gao, Y. S. Gao, F. M. Garay Walls, C. García, J. E. García Navarro, J. A. García Pascual, M. Garcia-Sciveres, R. W. Gardner, N. Garelli, V. Garonne, A. Gascon Bravo, K. Gasnikova, C. Gatti, A. Gaudiello, G. Gaudio, I. L. Gavrilenko, C. Gay, G. Gaycken, E. N. Gazis, C. N. P. Gee, J. Geisen, M. Geisen, M. P. Geisler, K. Gellerstedt, C. Gemme, M. H. Genest, C. Geng, S. Gentile, C. Gentsos, S. George, D. Gerbaudo, G. Geßner, S. Ghasemi, M. Ghneimat, B. Giacobbe, S. Giagu, N. Giangiacomi, P. Giannetti, S. M. Gibson, M. Gignac, M. Gilchriese, D. Gillberg, G. Gilles, D. M. Gingrich, M. P. Giordani, F. M. Giorgi, P. F. Giraud, P. Giromini, G. Giugliarelli, D. Giugni, F. Giuli, C. Giuliani, M. Giulini, B. K. Gjelsten, S. Gkaitatzis, I. Gkialas, E. L. Gkougkousis, P. Gkountoumis, L. K. Gladilin, C. Glasman, J. Glatzer, P. C. F. Glaysher, A. Glazov, M. Goblirsch-Kolb, J. Godlewski, S. Goldfarb, T. Golling, D. Golubkov, A. Gomes, R. Gonçalo, R. Goncalves Gama, J. Goncalves Pinto Firmino Da Costa, G. Gonella, L. Gonella, A. Gongadze, J. L. Gonski, S. González de la Hoz, S. Gonzalez-Sevilla, L. Goossens, P. A. Gorbounov, H. A. Gordon, I. Gorelov, B. Gorini, E. Gorini, A. Gorišek, A. T. Goshaw, C. Gössling, M. I. Gostkin, C. A. Gottardo, C. R. Goudet, D. Goujdami, A. G. Goussiou, N. Govender, E. Gozani, I. Grabowska-Bold, P. O. J. Gradin, J. Gramling, E. Gramstad, S. Grancagnolo, V. Gratchev, P. M. Gravila, C. Gray, H. M. Gray, Z. D. Greenwood, C. Grefe, K. Gregersen, I. M. Gregor, P. Grenier, K. Grevtsov, J. Griffiths, A. A. Grillo, K. Grimm, S. Grinstein, Ph. Gris, J.-F. Grivaz, S. Groh, E. Gross, J. Grosse-Knetter, G. C. Grossi, Z. J. Grout, A. Grummer, L. Guan, W. Guan, J. Guenther, F. Guescini, D. Guest, O. Gueta, B. Gui, E. Guido, T. Guillemin, S. Guindon, U. Gul, C. Gumpert, J. Guo, W. Guo, Y. Guo, R. Gupta, S. Gurbuz, G. Gustavino, B. J. Gutelman, P. Gutierrez, N. G. Gutierrez Ortiz, C. Gutschow, C. Guyot, M. P. Guzik, C. Gwenlan, C. B. Gwilliam, A. Haas, C. Haber, H. K. Hadavand, N. Haddad, A. Hadef, S. Hageböck, M. Hagihara, H. Hakobyan, M. Haleem, J. Haley, G. Halladjian, G. D. Hallewell, K. Hamacher, P. Hamal, K. Hamano, A. Hamilton, G. N. Hamity, P. G. Hamnett, L. Han, S. Han, K. Hanagaki, K. Hanawa, M. Hance, D. M. Handl, B. Haney, P. Hanke, J. B. Hansen, J. D. Hansen, M. C. Hansen, P. H. Hansen, K. Hara, A. S. Hard, T. Harenberg, F. Hariri, S. Harkusha, P. F. Harrison, N. M. Hartmann, Y. Hasegawa, A. Hasib, S. Hassani, S. Haug, R. Hauser, L. Hauswald, L. B. Havener, M. Havranek, C. M. Hawkes, R. J. Hawkings, D. Hayakawa, D. Hayden, C. P. Hays, J. M. Hays, H. S. Hayward, S. J. Haywood, S. J. Head, T. Heck, V. Hedberg, L. Heelan, S. Heer, K. K. Heidegger, S. Heim, T. Heim, B. Heinemann, J. J. Heinrich, L. Heinrich, C. Heinz, J. Hejbal, L. Helary, A. Held, S. Hellman, C. Helsens, R. C. W. Henderson, Y. Heng, S. Henkelmann, A. M. Henriques Correia, S. Henrot-Versille, G. H. Herbert, H. Herde, V. Herget, Y. Hernández Jiménez, H. Herr, G. Herten, R. Hertenberger, L. Hervas, T. C. Herwig, G. G. Hesketh, N. P. Hessey, J. W. Hetherly, S. Higashino, E. Higón-Rodriguez, K. Hildebrand, E. Hill, J. C. Hill, K. H. Hiller, S. J. Hillier, M. Hils, I. Hinchliffe, M. Hirose, D. Hirschbuehl, B. Hiti, O. Hladik, D. R. Hlaluku, X. Hoad, J. Hobbs, N. Hod, M. C. Hodgkinson, P. Hodgson, A. Hoecker, M. R. Hoeferkamp, F. Hoenig, D. Hohn, T. R. Holmes, M. Homann, S. Honda, T. Honda, T. M. Hong, B. H. Hooberman, W. H. Hopkins, Y. Horii, A. J. Horton, J-Y. Hostachy, A. Hostiuc, S. Hou, A. Hoummada, J. Howarth, J. Hoya, M. Hrabovsky, J. Hrdinka, I. Hristova, J. Hrivnac, T. Hryn’ova, A. Hrynevich, P. J. Hsu, S.-C. Hsu, Q. Hu, S. Hu, Y. Huang, Z. Hubacek, F. Hubaut, F. Huegging, T. B. Huffman, E. W. Hughes, M. Huhtinen, R. F. H. Hunter, P. Huo, N. Huseynov, J. Huston, J. Huth, R. Hyneman, G. Iacobucci, G. Iakovidis, I. Ibragimov, L. Iconomidou-Fayard, Z. Idrissi, P. Iengo, O. Igonkina, T. Iizawa, Y. Ikegami, M. Ikeno, Y. Ilchenko, D. Iliadis, N. Ilic, F. Iltzsche, G. Introzzi, P. Ioannou, M. Iodice, K. Iordanidou, V. Ippolito, M. F. Isacson, N. Ishijima, M. Ishino, M. Ishitsuka, C. Issever, S. Istin, F. Ito, J. M. Iturbe Ponce, R. Iuppa, H. Iwasaki, J. M. Izen, V. Izzo, S. Jabbar, P. Jackson, R. M. Jacobs, V. Jain, K. B. Jakobi, K. Jakobs, S. Jakobsen, T. Jakoubek, D. O. Jamin, D. K. Jana, R. Jansky, J. Janssen, M. Janus, P. A. Janus, G. Jarlskog, N. Javadov, T. Javůrek, M. Javurkova, F. Jeanneau, L. Jeanty, J. Jejelava, A. Jelinskas, P. Jenni, C. Jeske, S. Jézéquel, H. Ji, J. Jia, H. Jiang, Y. Jiang, Z. Jiang, S. Jiggins, J. Jimenez Pena, S. Jin, A. Jinaru, O. Jinnouchi, H. Jivan, P. Johansson, K. A. Johns, C. A. Johnson, W. J. Johnson, K. Jon-And, R. W. L. Jones, S. D. Jones, S. Jones, T. J. Jones, J. Jongmanns, P. M. Jorge, J. Jovicevic, X. Ju, A. Juste Rozas, M. K. Köhler, A. Kaczmarska, M. Kado, H. Kagan, M. Kagan, S. J. Kahn, T. Kaji, E. Kajomovitz, C. W. Kalderon, A. Kaluza, S. Kama, A. Kamenshchikov, N. Kanaya, L. Kanjir, V. A. Kantserov, J. Kanzaki, B. Kaplan, L. S. Kaplan, D. Kar, K. Karakostas, N. Karastathis, M. J. Kareem, E. Karentzos, S. N. Karpov, Z. M. Karpova, K. Karthik, V. Kartvelishvili, A. N. Karyukhin, K. Kasahara, L. Kashif, R. D. Kass, A. Kastanas, Y. Kataoka, C. Kato, A. Katre, J. Katzy, K. Kawade, K. Kawagoe, T. Kawamoto, G. Kawamura, E. F. Kay, V. F. Kazanin, R. Keeler, R. Kehoe, J. S. Keller, E. Kellermann, J. J. Kempster, J Kendrick, H. Keoshkerian, O. Kepka, B. P. Kerševan, S. Kersten, R. A. Keyes, M. Khader, F. Khalil-zada, A. Khanov, A. G. Kharlamov, T. Kharlamova, A. Khodinov, T. J. Khoo, V. Khovanskiy, E. Khramov, J. Khubua, S. Kido, C. R. Kilby, H. Y. Kim, S. H. Kim, Y. K. Kim, N. Kimura, O. M. Kind, B. T. King, D. Kirchmeier, J. Kirk, A. E. Kiryunin, T. Kishimoto, D. Kisielewska, V. Kitali, O. Kivernyk, E. Kladiva, T. Klapdor-Kleingrothaus, M. H. Klein, M. Klein, U. Klein, K. Kleinknecht, P. Klimek, A. Klimentov, R. Klingenberg, T. Klingl, T. Klioutchnikova, F. F. Klitzner, E.-E. Kluge, P. Kluit, S. Kluth, E. Kneringer, E. B. F. G. Knoops, A. Knue, A. Kobayashi, D. Kobayashi, T. Kobayashi, M. Kobel, M. Kocian, P. Kodys, T. Koffas, E. Koffeman, N. M. Köhler, T. Koi, M. Kolb, I. Koletsou, T. Kondo, N. Kondrashova, K. Köneke, A. C. König, T. Kono, R. Konoplich, N. Konstantinidis, B. Konya, R. Kopeliansky, S. Koperny, A. K. Kopp, K. Korcyl, K. Kordas, A. Korn, A. A. Korol, I. Korolkov, E. V. Korolkova, O. Kortner, S. Kortner, T. Kosek, V. V. Kostyukhin, A. Kotwal, A. Koulouris, A. Kourkoumeli-Charalampidi, C. Kourkoumelis, E. Kourlitis, V. Kouskoura, A. B. Kowalewska, R. Kowalewski, T. Z. Kowalski, C. Kozakai, W. Kozanecki, A. S. Kozhin, V. A. Kramarenko, G. Kramberger, D. Krasnopevtsev, M. W. Krasny, A. Krasznahorkay, D. Krauss, J. A. Kremer, J. Kretzschmar, K. Kreutzfeldt, P. Krieger, K. Krizka, K. Kroeninger, H. Kroha, J. Kroll, J. Kroll, J. Kroseberg, J. Krstic, U. Kruchonak, H. Krüger, N. Krumnack, M. C. Kruse, T. Kubota, H. Kucuk, S. Kuday, J. T. Kuechler, S. Kuehn, A. Kugel, F. Kuger, T. Kuhl, V. Kukhtin, R. Kukla, Y. Kulchitsky, S. Kuleshov, Y. P. Kulinich, M. Kuna, T. Kunigo, A. Kupco, T. Kupfer, O. Kuprash, H. Kurashige, L. L. Kurchaninov, Y. A. Kurochkin, M. G. Kurth, E. S. Kuwertz, M. Kuze, J. Kvita, T. Kwan, D. Kyriazopoulos, A. La Rosa, J. L. La Rosa Navarro, L. La Rotonda, F. La Ruffa, C. Lacasta, F. Lacava, J. Lacey, D. P. J. Lack, H. Lacker, D. Lacour, E. Ladygin, R. Lafaye, B. Laforge, T. Lagouri, S. Lai, S. Lammers, W. Lampl, E. Lançon, U. Landgraf, M. P. J. Landon, M. C. Lanfermann, V. S. Lang, J. C. Lange, R. J. Langenberg, A. J. Lankford, F. Lanni, K. Lantzsch, A. Lanza, A. Lapertosa, S. Laplace, J. F. Laporte, T. Lari, F. Lasagni Manghi, M. Lassnig, T. S. Lau, P. Laurelli, W. Lavrijsen, A. T. Law, P. Laycock, T. Lazovich, M. Lazzaroni, B. Le, O. Le Dortz, E. Le Guirriec, E. P. Le Quilleuc, M. LeBlanc, T. LeCompte, F. Ledroit-Guillon, C. A. Lee, G. R. Lee, S. C. Lee, L. Lee, B. Lefebvre, G. Lefebvre, M. Lefebvre, F. Legger, C. Leggett, G. Lehmann Miotto, X. Lei, W. A. Leight, M. A. L. Leite, R. Leitner, D. Lellouch, B. Lemmer, K. J. C. Leney, T. Lenz, B. Lenzi, R. Leone, S. Leone, C. Leonidopoulos, G. Lerner, C. Leroy, R. Les, A. A. J. Lesage, C. G. Lester, M. Levchenko, J. Levêque, D. Levin, L. J. Levinson, M. Levy, D. Lewis, B. Li, Changqiao Li, H. Li, L. Li, Q. Li, Q. Li, S. Li, X. Li, Y. Li, Z. Liang, B. Liberti, A. Liblong, K. Lie, J. Liebal, W. Liebig, A. Limosani, C. Y. Lin, K. Lin, S. C. Lin, T. H. Lin, R. A. Linck, B. E. Lindquist, A. E. Lionti, E. Lipeles, A. Lipniacka, M. Lisovyi, T. M. Liss, A. Lister, A. M. Litke, B. Liu, H. Liu, H. Liu, J. K. K. Liu, J. Liu, J. B. Liu, K. Liu, L. Liu, M. Liu, Y. L. Liu, Y. Liu, M. Livan, A. Lleres, J. Llorente Merino, S. L. Lloyd, C. Y. Lo, F. Lo Sterzo, E. M. Lobodzinska, P. Loch, F. K. Loebinger, A. Loesle, K. M. Loew, T. Lohse, K. Lohwasser, M. Lokajicek, B. A. Long, J. D. Long, R. E. Long, L. Longo, K. A. Looper, J. A. Lopez, I. Lopez Paz, A. Lopez Solis, J. Lorenz, N. Lorenzo Martinez, M. Losada, P. J. Lösel, X. Lou, A. Lounis, J. Love, P. A. Love, H. Lu, N. Lu, Y. J. Lu, H. J. Lubatti, C. Luci, A. Lucotte, C. Luedtke, F. Luehring, W. Lukas, L. Luminari, O. Lundberg, B. Lund-Jensen, M. S. Lutz, P. M. Luzi, D. Lynn, R. Lysak, E. Lytken, F. Lyu, V. Lyubushkin, H. Ma, L. L. Ma, Y. Ma, G. Maccarrone, A. Macchiolo, C. M. Macdonald, B. Maček, J. Machado Miguens, D. Madaffari, R. Madar, W. F. Mader, A. Madsen, N. Madysa, J. Maeda, S. Maeland, T. Maeno, A. S. Maevskiy, V. Magerl, C. Maiani, C. Maidantchik, T. Maier, A. Maio, O. Majersky, S. Majewski, Y. Makida, N. Makovec, B. Malaescu, Pa. Malecki, V. P. Maleev, F. Malek, U. Mallik, D. Malon, C. Malone, S. Maltezos, S. Malyukov, J. Mamuzic, G. Mancini, I. Mandić, J. Maneira, L. Manhaes de Andrade Filho, J. Manjarres Ramos, K. H. Mankinen, A. Mann, A. Manousos, B. Mansoulie, J. D. Mansour, R. Mantifel, M. Mantoani, S. Manzoni, L. Mapelli, G. Marceca, L. March, L. Marchese, G. Marchiori, M. Marcisovsky, C. A. Marin Tobon, M. Marjanovic, D. E. Marley, F. Marroquim, S. P. Marsden, Z. Marshall, M. U. F Martensson, S. Marti-Garcia, C. B. Martin, T. A. Martin, V. J. Martin, B. Martin dit Latour, M. Martinez, V. I. Martinez Outschoorn, S. Martin-Haugh, V. S. Martoiu, A. C. Martyniuk, A. Marzin, L. Masetti, T. Mashimo, R. Mashinistov, J. Masik, A. L. Maslennikov, L. H. Mason, L. Massa, P. Mastrandrea, A. Mastroberardino, T. Masubuchi, P. Mättig, J. Maurer, S. J. Maxfield, D. A. Maximov, R. Mazini, I. Maznas, S. M. Mazza, N. C. Mc Fadden, G. Mc Goldrick, S. P. Mc Kee, A. McCarn, R. L. McCarthy, T. G. McCarthy, L. I. McClymont, E. F. McDonald, J. A. Mcfayden, G. Mchedlidze, S. J. McMahon, P. C. McNamara, C. J. McNicol, R. A. McPherson, S. Meehan, T. J. Megy, S. Mehlhase, A. Mehta, T. Meideck, K. Meier, B. Meirose, D. Melini, B. R. Mellado Garcia, J. D. Mellenthin, M. Melo, F. Meloni, A. Melzer, S. B. Menary, L. Meng, X. T. Meng, A. Mengarelli, S. Menke, E. Meoni, S. Mergelmeyer, C. Merlassino, P. Mermod, L. Merola, C. Meroni, F. S. Merritt, A. Messina, J. Metcalfe, A. S. Mete, C. Meyer, J-P. Meyer, J. Meyer, H. Meyer Zu Theenhausen, F. Miano, R. P. Middleton, S. Miglioranzi, L. Mijović, G. Mikenberg, M. Mikestikova, M. Mikuž, M. Milesi, A. Milic, D. A. Millar, D. W. Miller, C. Mills, A. Milov, D. A. Milstead, A. A. Minaenko, Y. Minami, I. A. Minashvili, A. I. Mincer, B. Mindur, M. Mineev, Y. Minegishi, Y. Ming, L. M. Mir, A. Mirto, K. P. Mistry, T. Mitani, J. Mitrevski, V. A. Mitsou, A. Miucci, P. S. Miyagawa, A. Mizukami, J. U. Mjörnmark, T. Mkrtchyan, M. Mlynarikova, T. Moa, K. Mochizuki, P. Mogg, S. Mohapatra, S. Molander, R. Moles-Valls, M. C. Mondragon, K. Mönig, J. Monk, E. Monnier, A. Montalbano, J. Montejo Berlingen, F. Monticelli, S. Monzani, R. W. Moore, N. Morange, D. Moreno, M. Moreno Llácer, P. Morettini, S. Morgenstern, D. Mori, T. Mori, M. Morii, M. Morinaga, V. Morisbak, A. K. Morley, G. Mornacchi, J. D. Morris, L. Morvaj, P. Moschovakos, M. Mosidze, H. J. Moss, J. Moss, K. Motohashi, R. Mount, E. Mountricha, E. J. W. Moyse, S. Muanza, F. Mueller, J. Mueller, R. S. P. Mueller, D. Muenstermann, P. Mullen, G. A. Mullier, F. J. Munoz Sanchez, W. J. Murray, H. Musheghyan, M. Muškinja, A. G. Myagkov, M. Myska, B. P. Nachman, O. Nackenhorst, K. Nagai, R. Nagai, K. Nagano, Y. Nagasaka, K. Nagata, M. Nagel, E. Nagy, A. M. Nairz, Y. Nakahama, K. Nakamura, T. Nakamura, I. Nakano, R. F. Naranjo Garcia, R. Narayan, D. I. Narrias Villar, I. Naryshkin, T. Naumann, G. Navarro, R. Nayyar, H. A. Neal, P. Yu. Nechaeva, T. J. Neep, A. Negri, M. Negrini, S. Nektarijevic, C. Nellist, A. Nelson, M. E. Nelson, S. Nemecek, P. Nemethy, M. Nessi, M. S. Neubauer, M. Neumann, P. R. Newman, T. Y. Ng, Y. S. Ng, T. Nguyen Manh, R. B. Nickerson, R. Nicolaidou, J. Nielsen, N. Nikiforou, V. Nikolaenko, I. Nikolic-Audit, K. Nikolopoulos, P. Nilsson, Y. Ninomiya, A. Nisati, N. Nishu, R. Nisius, I. Nitsche, T. Nitta, T. Nobe, Y. Noguchi, M. Nomachi, I. Nomidis, M. A. Nomura, T. Nooney, M. Nordberg, N. Norjoharuddeen, O. Novgorodova, M. Nozaki, L. Nozka, K. Ntekas, E. Nurse, F. Nuti, K. O’connor, D. C. O’Neil, A. A. O’Rourke, V. O’Shea, F. G. Oakham, H. Oberlack, T. Obermann, J. Ocariz, A. Ochi, I. Ochoa, J. P. Ochoa-Ricoux, S. Oda, S. Odaka, A. Oh, S. H. Oh, C. C. Ohm, H. Ohman, H. Oide, H. Okawa, Y. Okumura, T. Okuyama, A. Olariu, L. F. Oleiro Seabra, S. A. Olivares Pino, D. Oliveira Damazio, M. J. R. Olsson, A. Olszewski, J. Olszowska, A. Onofre, K. Onogi, P. U. E. Onyisi, H. Oppen, M. J. Oreglia, Y. Oren, D. Orestano, N. Orlando, R. S. Orr, B. Osculati, R. Ospanov, G. Otero y Garzon, H. Otono, M. Ouchrif, F. Ould-Saada, A. Ouraou, K. P. Oussoren, Q. Ouyang, M. Owen, R. E. Owen, V. E. Ozcan, N. Ozturk, K. Pachal, A. Pacheco Pages, L. Pacheco Rodriguez, C. Padilla Aranda, S. Pagan Griso, M. Paganini, F. Paige, G. Palacino, S. Palazzo, S. Palestini, M. Palka, D. Pallin, E. St. Panagiotopoulou, I. Panagoulias, C. E. Pandini, J. G. Panduro Vazquez, P. Pani, S. Panitkin, D. Pantea, L. Paolozzi, Th. D. Papadopoulou, K. Papageorgiou, A. Paramonov, D. Paredes Hernandez, A. J. Parker, M. A. Parker, K. A. Parker, F. Parodi, J. A. Parsons, U. Parzefall, V. R. Pascuzzi, J. M. Pasner, E. Pasqualucci, S. Passaggio, Fr. Pastore, S. Pataraia, J. R. Pater, T. Pauly, B. Pearson, S. Pedraza Lopez, R. Pedro, S. V. Peleganchuk, O. Penc, C. Peng, H. Peng, J. Penwell, B. S. Peralva, M. M. Perego, D. V. Perepelitsa, F. Peri, L. Perini, H. Pernegger, S. Perrella, R. Peschke, V. D. Peshekhonov, K. Peters, R. F. Y. Peters, B. A. Petersen, T. C. Petersen, E. Petit, A. Petridis, C. Petridou, P. Petroff, E. Petrolo, M. Petrov, F. Petrucci, N. E. Pettersson, A. Peyaud, R. Pezoa, F. H. Phillips, P. W. Phillips, G. Piacquadio, E. Pianori, A. Picazio, M. A. Pickering, R. Piegaia, J. E. Pilcher, A. D. Pilkington, M. Pinamonti, J. L. Pinfold, H. Pirumov, M. Pitt, L. Plazak, M.-A. Pleier, V. Pleskot, E. Plotnikova, D. Pluth, P. Podberezko, R. Poettgen, R. Poggi, L. Poggioli, I. Pogrebnyak, D. Pohl, I. Pokharel, G. Polesello, A. Poley, A. Policicchio, R. Polifka, A. Polini, C. S. Pollard, V. Polychronakos, K. Pommès, D. Ponomarenko, L. Pontecorvo, G. A. Popeneciu, D. M. Portillo Quintero, S. Pospisil, K. Potamianos, I. N. Potrap, C. J. Potter, H. Potti, T. Poulsen, J. Poveda, M. E. Pozo Astigarraga, P. Pralavorio, A. Pranko, S. Prell, D. Price, M. Primavera, S. Prince, N. Proklova, K. Prokofiev, F. Prokoshin, S. Protopopescu, J. Proudfoot, M. Przybycien, A. Puri, P. Puzo, J. Qian, G. Qin, Y. Qin, A. Quadt, M. Queitsch-Maitland, D. Quilty, S. Raddum, V. Radeka, V. Radescu, S. K. Radhakrishnan, P. Radloff, P. Rados, F. Ragusa, G. Rahal, J. A. Raine, S. Rajagopalan, C. Rangel-Smith, T. Rashid, S. Raspopov, M. G. Ratti, D. M. Rauch, F. Rauscher, S. Rave, I. Ravinovich, J. H. Rawling, M. Raymond, A. L. Read, N. P. Readioff, M. Reale, D. M. Rebuzzi, A. Redelbach, G. Redlinger, R. Reece, R. G. Reed, K. Reeves, L. Rehnisch, J. Reichert, A. Reiss, C. Rembser, H. Ren, M. Rescigno, S. Resconi, E. D. Resseguie, S. Rettie, E. Reynolds, O. L. Rezanova, P. Reznicek, R. Rezvani, R. Richter, S. Richter, E. Richter-Was, O. Ricken, M. Ridel, P. Rieck, C. J. Riegel, J. Rieger, O. Rifki, M. Rijssenbeek, A. Rimoldi, M. Rimoldi, L. Rinaldi, G. Ripellino, B. Ristić, E. Ritsch, I. Riu, F. Rizatdinova, E. Rizvi, C. Rizzi, R. T. Roberts, S. H. Robertson, A. Robichaud-Veronneau, D. Robinson, J. E. M. Robinson, A. Robson, E. Rocco, C. Roda, Y. Rodina, S. Rodriguez Bosca, A. Rodriguez Perez, D. Rodriguez Rodriguez, S. Roe, C. S. Rogan, O. Røhne, J. Roloff, A. Romaniouk, M. Romano, S. M. Romano Saez, E. Romero Adam, N. Rompotis, M. Ronzani, L. Roos, S. Rosati, K. Rosbach, P. Rose, N.-A. Rosien, E. Rossi, L. P. Rossi, J. H. N. Rosten, R. Rosten, M. Rotaru, J. Rothberg, D. Rousseau, A. Rozanov, Y. Rozen, X. Ruan, F. Rubbo, F. Rühr, A. Ruiz-Martinez, Z. Rurikova, N. A. Rusakovich, H. L. Russell, J. P. Rutherfoord, N. Ruthmann, E. M. Rüttinger, Y. F. Ryabov, M. Rybar, G. Rybkin, S. Ryu, A. Ryzhov, G. F. Rzehorz, A. F. Saavedra, G. Sabato, S. Sacerdoti, H. F-W. Sadrozinski, R. Sadykov, F. Safai Tehrani, P. Saha, M. Sahinsoy, M. Saimpert, M. Saito, T. Saito, H. Sakamoto, Y. Sakurai, G. Salamanna, J. E. Salazar Loyola, D. Salek, P. H. Sales De Bruin, D. Salihagic, A. Salnikov, J. Salt, D. Salvatore, F. Salvatore, A. Salvucci, A. Salzburger, D. Sammel, D. Sampsonidis, D. Sampsonidou, J. Sánchez, V. Sanchez Martinez, A. Sanchez Pineda, H. Sandaker, R. L. Sandbach, C. O. Sander, M. Sandhoff, C. Sandoval, D. P. C. Sankey, M. Sannino, Y. Sano, A. Sansoni, C. Santoni, H. Santos, I. Santoyo Castillo, A. Sapronov, J. G. Saraiva, B. Sarrazin, O. Sasaki, K. Sato, E. Sauvan, G. Savage, P. Savard, N. Savic, C. Sawyer, L. Sawyer, J. Saxon, C. Sbarra, A. Sbrizzi, T. Scanlon, D. A. Scannicchio, J. Schaarschmidt, P. Schacht, B. M. Schachtner, D. Schaefer, L. Schaefer, R. Schaefer, J. Schaeffer, S. Schaepe, S. Schaetzel, U. Schäfer, A. C. Schaffer, D. Schaile, R. D. Schamberger, V. A. Schegelsky, D. Scheirich, M. Schernau, C. Schiavi, S. Schier, L. K. Schildgen, C. Schillo, M. Schioppa, S. Schlenker, K. R. Schmidt-Sommerfeld, K. Schmieden, C. Schmitt, S. Schmitt, S. Schmitz, U. Schnoor, L. Schoeffel, A. Schoening, B. D. Schoenrock, E. Schopf, M. Schott, J. F. P. Schouwenberg, J. Schovancova, S. Schramm, N. Schuh, A. Schulte, M. J. Schultens, H.-C. Schultz-Coulon, H. Schulz, M. Schumacher, B. A. Schumm, Ph. Schune, A. Schwartzman, T. A. Schwarz, H. Schweiger, Ph. Schwemling, R. Schwienhorst, J. Schwindling, A. Sciandra, G. Sciolla, M. Scornajenghi, F. Scuri, F. Scutti, J. Searcy, P. Seema, S. C. Seidel, A. Seiden, J. M. Seixas, G. Sekhniaidze, K. Sekhon, S. J. Sekula, N. Semprini-Cesari, S. Senkin, C. Serfon, L. Serin, L. Serkin, M. Sessa, R. Seuster, H. Severini, T. Sfiligoj, F. Sforza, A. Sfyrla, E. Shabalina, N. W. Shaikh, L. Y. Shan, R. Shang, J. T. Shank, M. Shapiro, P. B. Shatalov, K. Shaw, S. M. Shaw, A. Shcherbakova, C. Y. Shehu, Y. Shen, N. Sherafati, A. D. Sherman, P. Sherwood, L. Shi, S. Shimizu, C. O. Shimmin, M. Shimojima, I. P. J. Shipsey, S. Shirabe, M. Shiyakova, J. Shlomi, A. Shmeleva, D. Shoaleh Saadi, M. J. Shochet, S. Shojaii, D. R. Shope, S. Shrestha, E. Shulga, M. A. Shupe, P. Sicho, A. M. Sickles, P. E. Sidebo, E. Sideras Haddad, O. Sidiropoulou, A. Sidoti, F. Siegert, Dj. Sijacki, J. Silva, S. B. Silverstein, V. Simak, L. Simic, S. Simion, E. Simioni, B. Simmons, M. Simon, P. Sinervo, N. B. Sinev, M. Sioli, G. Siragusa, I. Siral, S. Yu. Sivoklokov, J. Sjölin, M. B. Skinner, P. Skubic, M. Slater, T. Slavicek, M. Slawinska, K. Sliwa, R. Slovak, V. Smakhtin, B. H. Smart, J. Smiesko, N. Smirnov, S. Yu. Smirnov, Y. Smirnov, L. N. Smirnova, O. Smirnova, J. W. Smith, M. N. K. Smith, R. W. Smith, M. Smizanska, K. Smolek, A. A. Snesarev, I. M. Snyder, S. Snyder, R. Sobie, F. Socher, A. Soffer, A. Søgaard, D. A. Soh, G. Sokhrannyi, C. A. Solans Sanchez, M. Solar, E. Yu. Soldatov, U. Soldevila, A. A. Solodkov, A. Soloshenko, O. V. Solovyanov, V. Solovyev, P. Sommer, H. Son, A. Sopczak, D. Sosa, C. L. Sotiropoulou, S. Sottocornola, R. Soualah, A. M. Soukharev, D. South, B. C. Sowden, S. Spagnolo, M. Spalla, M. Spangenberg, F. Spanò, D. Sperlich, F. Spettel, T. M. Spieker, R. Spighi, G. Spigo, L. A. Spiller, M. Spousta, R. D. St. Denis, A. Stabile, R. Stamen, S. Stamm, E. Stanecka, R. W. Stanek, C. Stanescu, M. M. Stanitzki, B. S. Stapf, S. Stapnes, E. A. Starchenko, G. H. Stark, J. Stark, S. H Stark, P. Staroba, P. Starovoitov, S. Stärz, R. Staszewski, M. Stegler, P. Steinberg, B. Stelzer, H. J. Stelzer, O. Stelzer-Chilton, H. Stenzel, T. J. Stevenson, G. A. Stewart, M. C. Stockton, M. Stoebe, G. Stoicea, P. Stolte, S. Stonjek, A. R. Stradling, A. Straessner, M. E. Stramaglia, J. Strandberg, S. Strandberg, M. Strauss, P. Strizenec, R. Ströhmer, D. M. Strom, R. Stroynowski, A. Strubig, S. A. Stucci, B. Stugu, N. A. Styles, D. Su, J. Su, S. Suchek, Y. Sugaya, M. Suk, V. V. Sulin, D M S Sultan, S. Sultansoy, T. Sumida, S. Sun, X. Sun, K. Suruliz, C. J. E. Suster, M. R. Sutton, S. Suzuki, M. Svatos, M. Swiatlowski, S. P. Swift, I. Sykora, T. Sykora, D. Ta, K. Tackmann, J. Taenzer, A. Taffard, R. Tafirout, E. Tahirovic, N. Taiblum, H. Takai, R. Takashima, E. H. Takasugi, K. Takeda, T. Takeshita, Y. Takubo, M. Talby, A. A. Talyshev, J. Tanaka, M. Tanaka, R. Tanaka, S. Tanaka, R. Tanioka, B. B. Tannenwald, S. Tapia Araya, S. Tapprogge, S. Tarem, G. F. Tartarelli, P. Tas, M. Tasevsky, T. Tashiro, E. Tassi, A. Tavares Delgado, Y. Tayalati, A. C. Taylor, A. J. Taylor, G. N. Taylor, P. T. E. Taylor, W. Taylor, P. Teixeira-Dias, D. Temple, H. Ten Kate, P. K. Teng, J. J. Teoh, F. Tepel, S. Terada, K. Terashi, J. Terron, S. Terzo, M. Testa, R. J. Teuscher, S. J. Thais, T. Theveneaux-Pelzer, F. Thiele, J. P. Thomas, J. Thomas-Wilsker, P. D. Thompson, A. S. Thompson, L. A. Thomsen, E. Thomson, Y. Tian, M. J. Tibbetts, R. E. Ticse Torres, V. O. Tikhomirov, Yu. A. Tikhonov, S. Timoshenko, P. Tipton, S. Tisserant, K. Todome, S. Todorova-Nova, S. Todt, J. Tojo, S. Tokár, K. Tokushuku, E. Tolley, L. Tomlinson, M. Tomoto, L. Tompkins, K. Toms, B. Tong, P. Tornambe, E. Torrence, H. Torres, E. Torró Pastor, J. Toth, F. Touchard, D. R. Tovey, C. J. Treado, T. Trefzger, F. Tresoldi, A. Tricoli, I. M. Trigger, S. Trincaz-Duvoid, M. F. Tripiana, W. Trischuk, B. Trocmé, A. Trofymov, C. Troncon, M. Trottier-McDonald, M. Trovatelli, L. Truong, M. Trzebinski, A. Trzupek, K. W. Tsang, J. C-L. Tseng, P. V. Tsiareshka, N. Tsirintanis, S. Tsiskaridze, V. Tsiskaridze, E. G. Tskhadadze, I. I. Tsukerman, V. Tsulaia, S. Tsuno, D. Tsybychev, Y. Tu, A. Tudorache, V. Tudorache, T. T. Tulbure, A. N. Tuna, S. Turchikhin, D. Turgeman, I. Turk Cakir, R. Turra, P. M. Tuts, G. Ucchielli, I. Ueda, M. Ughetto, F. Ukegawa, G. Unal, A. Undrus, G. Unel, F. C. Ungaro, Y. Unno, K. Uno, C. Unverdorben, J. Urban, P. Urquijo, P. Urrejola, G. Usai, J. Usui, L. Vacavant, V. Vacek, B. Vachon, K. O. H. Vadla, A. Vaidya, C. Valderanis, E. Valdes Santurio, M. Valente, S. Valentinetti, A. Valero, L. Valéry, S. Valkar, A. Vallier, J. A. Valls Ferrer, W. Van Den Wollenberg, H. van der Graaf, P. van Gemmeren, J. Van Nieuwkoop, I. van Vulpen, M. C. van Woerden, M. Vanadia, W. Vandelli, A. Vaniachine, P. Vankov, G. Vardanyan, R. Vari, E. W. Varnes, C. Varni, T. Varol, D. Varouchas, A. Vartapetian, K. E. Varvell, J. G. Vasquez, G. A. Vasquez, F. Vazeille, D. Vazquez Furelos, T. Vazquez Schroeder, J. Veatch, V. Veeraraghavan, L. M. Veloce, F. Veloso, S. Veneziano, A. Ventura, M. Venturi, N. Venturi, A. Venturini, V. Vercesi, M. Verducci, W. Verkerke, A. T. Vermeulen, J. C. Vermeulen, M. C. Vetterli, N. Viaux Maira, O. Viazlo, I. Vichou, T. Vickey, O. E. Vickey Boeriu, G. H. A. Viehhauser, S. Viel, L. Vigani, M. Villa, M. Villaplana Perez, E. Vilucchi, M. G. Vincter, V. B. Vinogradov, A. Vishwakarma, C. Vittori, I. Vivarelli, S. Vlachos, M. Vogel, P. Vokac, G. Volpi, H. von der Schmitt, E. von Toerne, V. Vorobel, K. Vorobev, M. Vos, R. Voss, J. H. Vossebeld, N. Vranjes, M. Vranjes Milosavljevic, V. Vrba, M. Vreeswijk, R. Vuillermet, I. Vukotic, P. Wagner, W. Wagner, J. Wagner-Kuhr, H. Wahlberg, S. Wahrmund, K. Wakamiya, J. Walder, R. Walker, W. Walkowiak, V. Wallangen, C. Wang, C. Wang, F. Wang, H. Wang, H. Wang, J. Wang, J. Wang, Q. Wang, R.-J. Wang, R. Wang, S. M. Wang, T. Wang, W. Wang, W. Wang, Z. Wang, C. Wanotayaroj, A. Warburton, C. P. Ward, D. R. Wardrope, A. Washbrook, P. M. Watkins, A. T. Watson, M. F. Watson, G. Watts, S. Watts, B. M. Waugh, A. F. Webb, S. Webb, M. S. Weber, S. M. Weber, S. W. Weber, S. A. Weber, J. S. Webster, A. R. Weidberg, B. Weinert, J. Weingarten, M. Weirich, C. Weiser, H. Weits, P. S. Wells, T. Wenaus, T. Wengler, S. Wenig, N. Wermes, M. D. Werner, P. Werner, M. Wessels, T. D. Weston, K. Whalen, N. L. Whallon, A. M. Wharton, A. S. White, A. White, M. J. White, R. White, D. Whiteson, B. W. Whitmore, F. J. Wickens, W. Wiedenmann, M. Wielers, C. Wiglesworth, L. A. M. Wiik-Fuchs, A. Wildauer, F. Wilk, H. G. Wilkens, H. H. Williams, S. Williams, C. Willis, S. Willocq, J. A. Wilson, I. Wingerter-Seez, E. Winkels, F. Winklmeier, O. J. Winston, B. T. Winter, M. Wittgen, M. Wobisch, A. Wolf, T. M. H. Wolf, R. Wolff, M. W. Wolter, H. Wolters, V. W. S. Wong, N. L. Woods, S. D. Worm, B. K. Wosiek, J. Wotschack, K. W. Wozniak, M. Wu, S. L. Wu, X. Wu, Y. Wu, T. R. Wyatt, B. M. Wynne, S. Xella, Z. Xi, L. Xia, D. Xu, L. Xu, T. Xu, W. Xu, B. Yabsley, S. Yacoob, D. Yamaguchi, Y. Yamaguchi, A. Yamamoto, S. Yamamoto, T. Yamanaka, F. Yamane, M. Yamatani, T. Yamazaki, Y. Yamazaki, Z. Yan, H. Yang, H. Yang, Y. Yang, Z. Yang, W-M. Yao, Y. C. Yap, Y. Yasu, E. Yatsenko, K. H. Yau Wong, J. Ye, S. Ye, I. Yeletskikh, E. Yigitbasi, E. Yildirim, K. Yorita, K. Yoshihara, C. Young, C. J. S. Young, J. Yu, J. Yu, S. P. Y. Yuen, I. Yusuff, B. Zabinski, G. Zacharis, R. Zaidan, A. M. Zaitsev, N. Zakharchuk, J. Zalieckas, A. Zaman, S. Zambito, D. Zanzi, C. Zeitnitz, G. Zemaityte, A. Zemla, J. C. Zeng, Q. Zeng, O. Zenin, T. Ženiš, D. Zerwas, D. Zhang, D. Zhang, F. Zhang, G. Zhang, H. Zhang, J. Zhang, L. Zhang, L. Zhang, M. Zhang, P. Zhang, R. Zhang, R. Zhang, X. Zhang, Y. Zhang, Z. Zhang, X. Zhao, Y. Zhao, Z. Zhao, A. Zhemchugov, B. Zhou, C. Zhou, L. Zhou, M. Zhou, M. Zhou, N. Zhou, Y. Zhou, C. G. Zhu, H. Zhu, J. Zhu, Y. Zhu, X. Zhuang, K. Zhukov, A. Zibell, D. Zieminska, N. I. Zimine, C. Zimmermann, S. Zimmermann, Z. Zinonos, M. Zinser, M. Ziolkowski, L. Živković, G. Zobernig, A. Zoccoli, R. Zou, M. zur Nedden, L. Zwalinski, ATLAS Collaboration

**Affiliations:** 1 Department of Physics, University of Adelaide, Adelaide, Australia; 2 Physics Department, SUNY Albany, Albany, NY USA; 3 Department of Physics, University of Alberta, Edmonton, AB Canada; 4 Department of Physics, Ankara University, Ankara, Turkey; 5 Istanbul Aydin University, Istanbul, Turkey; 6 Division of Physics, TOBB University of Economics and Technology, Ankara, Turkey; 7 LAPP, CNRS/IN2P3 and Université Savoie Mont Blanc, Annecy-le-Vieux, France; 8 High Energy Physics Division, Argonne National Laboratory, Argonne, IL USA; 9 Department of Physics, University of Arizona, Tucson, AZ USA; 10 Department of Physics, The University of Texas at Arlington, Arlington, TX USA; 11 Physics Department, National and Kapodistrian University of Athens, Athens, Greece; 12 Physics Department, National Technical University of Athens, Zografou, Greece; 13 Department of Physics, The University of Texas at Austin, Austin, TX USA; 14 Institute of Physics, Azerbaijan Academy of Sciences, Baku, Azerbaijan; 15 Institut de Física d’Altes Energies (IFAE), The Barcelona Institute of Science and Technology, Barcelona, Spain; 16 Institute of Physics, University of Belgrade, Belgrade, Serbia; 17 Department for Physics and Technology, University of Bergen, Bergen, Norway; 18 Physics Division, Lawrence Berkeley National Laboratory and University of California, Berkeley, CA USA; 19 Department of Physics, Humboldt University, Berlin, Germany; 20 Albert Einstein Center for Fundamental Physics, Laboratory for High Energy Physics, University of Bern, Bern, Switzerland; 21 School of Physics and Astronomy, University of Birmingham, Birmingham, UK; 22 Department of Physics, Bogazici University, Istanbul, Turkey; 23 Department of Physics Engineering, Gaziantep University, Gaziantep, Turkey; 24 Faculty of Engineering and Natural Sciences, Istanbul Bilgi University, Istanbul, Turkey; 25 Faculty of Engineering and Natural Sciences, Bahcesehir University, Istanbul, Turkey; 26 Centro de Investigaciones, Universidad Antonio Narino, Bogotá, Colombia; 27 INFN Sezione di Bologna, Bologna, Italy; 28 Dipartimento di Fisica e Astronomia, Università di Bologna, Bologna, Italy; 29 Physikalisches Institut, University of Bonn, Bonn, Germany; 30 Department of Physics, Boston University, Boston, MA USA; 31 Department of Physics, Brandeis University, Waltham, MA USA; 32 Universidade Federal do Rio De Janeiro COPPE/EE/IF, Rio de Janeiro, Brazil; 33 Electrical Circuits Department, Federal University of Juiz de Fora (UFJF), Juiz de Fora, Brazil; 34 Federal University of Sao Joao del Rei (UFSJ), Sao Joao del Rei, Brazil; 35 Instituto de Fisica, Universidade de Sao Paulo, São Paulo, Brazil; 36 Physics Department, Brookhaven National Laboratory, Upton, NY USA; 37 Transilvania University of Brasov, Brasov, Romania; 38 Horia Hulubei National Institute of Physics and Nuclear Engineering, Bucharest, Romania; 39 Department of Physics, Alexandru Ioan Cuza University of Iasi, Iasi, Romania; 40 Physics Department, National Institute for Research and Development of Isotopic and Molecular Technologies, Cluj-Napoca, Romania; 41 University Politehnica Bucharest, Bucharest, Romania; 42 West University in Timisoara, Timisoara, Romania; 43 Departamento de Física, Universidad de Buenos Aires, Buenos Aires, Argentina; 44 Cavendish Laboratory, University of Cambridge, Cambridge, UK; 45 Department of Physics, Carleton University, Ottawa, ON Canada; 46 CERN, Geneva, Switzerland; 47 Enrico Fermi Institute, University of Chicago, Chicago, IL USA; 48 Departamento de Física, Pontificia Universidad Católica de Chile, Santiago, Chile; 49 Departamento de Física, Universidad Técnica Federico Santa María, Valparaiso, Chile; 50 Institute of High Energy Physics, Chinese Academy of Sciences, Beijing, China; 51 Department of Physics, Nanjing University, Nanjing, Jiangsu China; 52 Physics Department, Tsinghua University, Beijing, 100084 China; 53 University of Chinese Academy of Science (UCAS), Beijing, China; 54 Department of Modern Physics and State Key Laboratory of Particle Detection and Electronics, University of Science and Technology of China, Hefei, Anhui China; 55 School of Physics, Shandong University, Jinan, Shandong China; 56 Department of Physics and Astronomy, Key Laboratory for Particle Physics, Astrophysics and Cosmology, Ministry of Education, Shanghai Key Laboratory for Particle Physics and Cosmology, Shanghai Jiao Tong University, Shanghai (also at PKU-CHEP), Shanghai, China; 57 Université Clermont Auvergne, CNRS/IN2P3, LPC, Clermont-Ferrand, France; 58 Nevis Laboratory, Columbia University, Irvington, NY USA; 59 Niels Bohr Institute, University of Copenhagen, Copenhagen, Denmark; 60 INFN Gruppo Collegato di Cosenza, Laboratori Nazionali di Frascati, Frascati, Italy; 61 Dipartimento di Fisica, Università della Calabria, Rende, Italy; 62 Faculty of Physics and Applied Computer Science, AGH University of Science and Technology, Kraków, Poland; 63 Marian Smoluchowski Institute of Physics, Jagiellonian University, Kraków, Poland; 64 Institute of Nuclear Physics, Polish Academy of Sciences, Kraków, Poland; 65 Physics Department, Southern Methodist University, Dallas, TX USA; 66 Physics Department, University of Texas at Dallas, Richardson, TX USA; 67 DESY, Hamburg and Zeuthen, Germany; 68 Lehrstuhl für Experimentelle Physik IV, Technische Universität Dortmund, Dortmund, Germany; 69 Institut für Kern- und Teilchenphysik, Technische Universität Dresden, Dresden, Germany; 70 Department of Physics, Duke University, Durham, NC USA; 71 SUPA-School of Physics and Astronomy, University of Edinburgh, Edinburgh, UK; 72 INFN e Laboratori Nazionali di Frascati, Frascati, Italy; 73 Fakultät für Mathematik und Physik, Albert-Ludwigs-Universität, Freiburg, Germany; 74 Departement de Physique Nucleaire et Corpusculaire, Université de Genève, Geneva, Switzerland; 75 INFN Sezione di Genova, Genoa, Italy; 76 Dipartimento di Fisica, Università di Genova, Genoa, Italy; 77 E. Andronikashvili Institute of Physics, Iv. Javakhishvili Tbilisi State University, Tbilisi, Georgia; 78 High Energy Physics Institute, Tbilisi State University, Tbilisi, Georgia; 79 II Physikalisches Institut, Justus-Liebig-Universität Giessen, Giessen, Germany; 80 SUPA-School of Physics and Astronomy, University of Glasgow, Glasgow, UK; 81 II Physikalisches Institut, Georg-August-Universität, Göttingen, Germany; 82 Laboratoire de Physique Subatomique et de Cosmologie, Université Grenoble-Alpes, CNRS/IN2P3, Grenoble, France; 83 Laboratory for Particle Physics and Cosmology, Harvard University, Cambridge, MA USA; 84 Kirchhoff-Institut für Physik, Ruprecht-Karls-Universität Heidelberg, Heidelberg, Germany; 85 Physikalisches Institut, Ruprecht-Karls-Universität Heidelberg, Heidelberg, Germany; 86 Faculty of Applied Information Science, Hiroshima Institute of Technology, Hiroshima, Japan; 87 Department of Physics, The Chinese University of Hong Kong, Shatin, NT Hong Kong; 88 Department of Physics, The University of Hong Kong, Hong Kong, China; 89 Department of Physics, Institute for Advanced Study, The Hong Kong University of Science and Technology, Clear Water Bay, Kowloon, Hong Kong, China; 90 Department of Physics, National Tsing Hua University, Hsinchu City, Taiwan; 91 Department of Physics, Indiana University, Bloomington, IN USA; 92 Institut für Astro- und Teilchenphysik, Leopold-Franzens-Universität, Innsbruck, Austria; 93 University of Iowa, Iowa City, IA USA; 94 Department of Physics and Astronomy, Iowa State University, Ames, IA USA; 95 Joint Institute for Nuclear Research, JINR Dubna, Dubna, Russia; 96 KEK, High Energy Accelerator Research Organization, Tsukuba, Japan; 97 Graduate School of Science, Kobe University, Kobe, Japan; 98 Faculty of Science, Kyoto University, Kyoto, Japan; 99 Kyoto University of Education, Kyoto, Japan; 100 Research Center for Advanced Particle Physics and Department of Physics, Kyushu University, Fukuoka, Japan; 101 Instituto de Física La Plata, Universidad Nacional de La Plata and CONICET, La Plata, Argentina; 102 Physics Department, Lancaster University, Lancaster, UK; 103 INFN Sezione di Lecce, Lecce, Italy; 104 Dipartimento di Matematica e Fisica, Università del Salento, Lecce, Italy; 105 Oliver Lodge Laboratory, University of Liverpool, Liverpool, UK; 106 Department of Experimental Particle Physics, Jožef Stefan Institute and Department of Physics, University of Ljubljana, Ljubljana, Slovenia; 107 School of Physics and Astronomy, Queen Mary University of London, London, UK; 108 Department of Physics, Royal Holloway University of London, Surrey, UK; 109 Department of Physics and Astronomy, University College London, London, UK; 110 Louisiana Tech University, Ruston, LA USA; 111 Laboratoire de Physique Nucléaire et de Hautes Energies, UPMC and Université Paris-Diderot and CNRS/IN2P3, Paris, France; 112 Fysiska institutionen, Lunds universitet, Lund, Sweden; 113 Departamento de Fisica Teorica C-15, Universidad Autonoma de Madrid, Madrid, Spain; 114 Institut für Physik, Universität Mainz, Mainz, Germany; 115 School of Physics and Astronomy, University of Manchester, Manchester, UK; 116 CPPM, Aix-Marseille Université and CNRS/IN2P3, Marseille, France; 117 Department of Physics, University of Massachusetts, Amherst, MA USA; 118 Department of Physics, McGill University, Montreal, QC Canada; 119 School of Physics, University of Melbourne, Victoria, Australia; 120 Department of Physics, The University of Michigan, Ann Arbor, MI USA; 121 Department of Physics and Astronomy, Michigan State University, East Lansing, MI USA; 122 INFN Sezione di Milano, Milan, Italy; 123 Dipartimento di Fisica, Università di Milano, Milan, Italy; 124 B.I. Stepanov Institute of Physics, National Academy of Sciences of Belarus, Minsk, Republic of Belarus; 125 Research Institute for Nuclear Problems of Byelorussian State University, Minsk, Republic of Belarus; 126 Group of Particle Physics, University of Montreal, Montreal, QC Canada; 127 P.N. Lebedev Physical Institute of the Russian Academy of Sciences, Moscow, Russia; 128 Institute for Theoretical and Experimental Physics (ITEP), Moscow, Russia; 129 National Research Nuclear University MEPhI, Moscow, Russia; 130 D.V. Skobeltsyn Institute of Nuclear Physics, M.V. Lomonosov Moscow State University, Moscow, Russia; 131 Fakultät für Physik, Ludwig-Maximilians-Universität München, Munich, Germany; 132 Max-Planck-Institut für Physik (Werner-Heisenberg-Institut), Munich, Germany; 133 Nagasaki Institute of Applied Science, Nagasaki, Japan; 134 Graduate School of Science and Kobayashi-Maskawa Institute, Nagoya University, Nagoya, Japan; 135 INFN Sezione di Napoli, Naples, Italy; 136 Dipartimento di Fisica, Università di Napoli, Naples, Italy; 137 Department of Physics and Astronomy, University of New Mexico, Albuquerque, NM USA; 138 Institute for Mathematics, Astrophysics and Particle Physics, Radboud University Nijmegen/Nikhef, Nijmegen, The Netherlands; 139 Nikhef National Institute for Subatomic Physics and University of Amsterdam, Amsterdam, The Netherlands; 140 Department of Physics, Northern Illinois University, DeKalb, IL USA; 141 Budker Institute of Nuclear Physics, SB RAS, Novosibirsk, Russia; 142 Department of Physics, New York University, New York, NY USA; 143 Ohio State University, Columbus, OH USA; 144 Faculty of Science, Okayama University, Okayama, Japan; 145 Homer L. Dodge Department of Physics and Astronomy, University of Oklahoma, Norman, OK USA; 146 Department of Physics, Oklahoma State University, Stillwater, OK USA; 147 Palacký University, RCPTM, Olomouc, Czech Republic; 148 Center for High Energy Physics, University of Oregon, Eugene, OR USA; 149 LAL, Univ. Paris-Sud, CNRS/IN2P3, Université Paris-Saclay, Orsay, France; 150 Graduate School of Science, Osaka University, Osaka, Japan; 151 Department of Physics, University of Oslo, Oslo, Norway; 152 Department of Physics, Oxford University, Oxford, UK; 153 INFN Sezione di Pavia, Pavia, Italy; 154 Dipartimento di Fisica, Università di Pavia, Pavia, Italy; 155 Department of Physics, University of Pennsylvania, Philadelphia, PA USA; 156 National Research Centre “Kurchatov Institute” B.P. Konstantinov Petersburg Nuclear Physics Institute, St. Petersburg, Russia; 157 INFN Sezione di Pisa, Pisa, Italy; 158 Dipartimento di Fisica E. Fermi, Università di Pisa, Pisa, Italy; 159 Department of Physics and Astronomy, University of Pittsburgh, Pittsburgh, PA USA; 160 Laboratório de Instrumentação e Física Experimental de Partículas-LIP, Lisbon, Portugal; 161 Faculdade de Ciências, Universidade de Lisboa, Lisbon, Portugal; 162 Department of Physics, University of Coimbra, Coimbra, Portugal; 163 Centro de Física Nuclear da Universidade de Lisboa, Lisbon, Portugal; 164 Departamento de Fisica, Universidade do Minho, Braga, Portugal; 165 Departamento de Fisica Teorica y del Cosmos, Universidad de Granada, Granada, Spain; 166 Dep Fisica and CEFITEC of Faculdade de Ciencias e Tecnologia, Universidade Nova de Lisboa, Caparica, Portugal; 167 Institute of Physics, Academy of Sciences of the Czech Republic, Prague, Czech Republic; 168 Czech Technical University in Prague, Prague, Czech Republic; 169 Faculty of Mathematics and Physics, Charles University, Prague, Czech Republic; 170 State Research Center Institute for High Energy Physics (Protvino), NRC KI, Protvino, Russia; 171 Particle Physics Department, Rutherford Appleton Laboratory, Didcot, UK; 172 INFN Sezione di Roma, Rome, Italy; 173 Dipartimento di Fisica, Sapienza Università di Roma, Rome, Italy; 174 INFN Sezione di Roma Tor Vergata, Rome, Italy; 175 Dipartimento di Fisica, Università di Roma Tor Vergata, Rome, Italy; 176 INFN Sezione di Roma Tre, Rome, Italy; 177 Dipartimento di Matematica e Fisica, Università Roma Tre, Rome, Italy; 178 Faculté des Sciences Ain Chock, Réseau Universitaire de Physique des Hautes Energies-Université Hassan II, Casablanca, Morocco; 179 Centre National de l’Energie des Sciences Techniques Nucleaires, Rabat, Morocco; 180 Faculté des Sciences Semlalia, Université Cadi Ayyad, LPHEA-Marrakech, Marrakech, Morocco; 181 Faculté des Sciences, Université Mohamed Premier and LPTPM, Oujda, Morocco; 182 Faculté des Sciences, Université Mohammed V, Rabat, Morocco; 183 DSM/IRFU (Institut de Recherches sur les Lois Fondamentales de l’Univers), CEA Saclay (Commissariat à l’Energie Atomique et aux Energies Alternatives), Gif-sur-Yvette, France; 184 Santa Cruz Institute for Particle Physics, University of California Santa Cruz, Santa Cruz, CA USA; 185 Department of Physics, University of Washington, Seattle, WA USA; 186 Department of Physics and Astronomy, University of Sheffield, Sheffield, UK; 187 Department of Physics, Shinshu University, Nagano, Japan; 188 Department Physik, Universität Siegen, Siegen, Germany; 189 Department of Physics, Simon Fraser University, Burnaby, BC Canada; 190 SLAC National Accelerator Laboratory, Stanford, CA USA; 191 Faculty of Mathematics, Physics and Informatics, Comenius University, Bratislava, Slovak Republic; 192 Department of Subnuclear Physics, Institute of Experimental Physics of the Slovak Academy of Sciences, Kosice, Slovak Republic; 193 Department of Physics, University of Cape Town, Cape Town, South Africa; 194 Department of Physics, University of Johannesburg, Johannesburg, South Africa; 195 School of Physics, University of the Witwatersrand, Johannesburg, South Africa; 196 Department of Physics, Stockholm University, Stockholm, Sweden; 197 The Oskar Klein Centre, Stockholm, Sweden; 198 Physics Department, Royal Institute of Technology, Stockholm, Sweden; 199 Departments of Physics and Astronomy and Chemistry, Stony Brook University, Stony Brook, NY USA; 200 Department of Physics and Astronomy, University of Sussex, Brighton, UK; 201 School of Physics, University of Sydney, Sydney, Australia; 202 Institute of Physics, Academia Sinica, Taipei, Taiwan; 203 Department of Physics, Technion: Israel Institute of Technology, Haifa, Israel; 204 Raymond and Beverly Sackler School of Physics and Astronomy, Tel Aviv University, Tel Aviv, Israel; 205 Department of Physics, Aristotle University of Thessaloniki, Thessaloníki, Greece; 206 International Center for Elementary Particle Physics and Department of Physics, The University of Tokyo, Tokyo, Japan; 207 Graduate School of Science and Technology, Tokyo Metropolitan University, Tokyo, Japan; 208 Department of Physics, Tokyo Institute of Technology, Tokyo, Japan; 209 Tomsk State University, Tomsk, Russia; 210 Department of Physics, University of Toronto, Toronto, ON Canada; 211 INFN-TIFPA, Trento, Italy; 212 University of Trento, Trento, Italy; 213 TRIUMF, Vancouver, BC Canada; 214 Department of Physics and Astronomy, York University, Toronto, ON Canada; 215 Faculty of Pure and Applied Sciences, and Center for Integrated Research in Fundamental Science and Engineering, University of Tsukuba, Tsukuba, Japan; 216 Department of Physics and Astronomy, Tufts University, Medford, MA USA; 217 Department of Physics and Astronomy, University of California Irvine, Irvine, CA USA; 218 INFN Gruppo Collegato di Udine, Sezione di Trieste, Udine, Italy; 219 ICTP, Trieste, Italy; 220 Dipartimento di Chimica, Fisica e Ambiente, Università di Udine, Udine, Italy; 221 Department of Physics and Astronomy, University of Uppsala, Uppsala, Sweden; 222 Department of Physics, University of Illinois, Urbana, IL USA; 223 Instituto de Fisica Corpuscular (IFIC), Centro Mixto Universidad de Valencia - CSIC, Valencia, Spain; 224 Department of Physics, University of British Columbia, Vancouver, BC Canada; 225 Department of Physics and Astronomy, University of Victoria, Victoria, BC Canada; 226 Department of Physics, University of Warwick, Coventry, UK; 227 Waseda University, Tokyo, Japan; 228 Department of Particle Physics, The Weizmann Institute of Science, Rehovot, Israel; 229 Department of Physics, University of Wisconsin, Madison, WI USA; 230 Fakultät für Physik und Astronomie, Julius-Maximilians-Universität, Würzburg, Germany; 231 Fakultät für Mathematik und Naturwissenschaften, Fachgruppe Physik, Bergische Universität Wuppertal, Wuppertal, Germany; 232 Department of Physics, Yale University, New Haven, CT USA; 233 Yerevan Physics Institute, Yerevan, Armenia; 234 Centre de Calcul de l’Institut National de Physique Nucléaire et de Physique des Particules (IN2P3), Villeurbanne, France; 235 Academia Sinica Grid Computing, Institute of Physics, Academia Sinica, Taipei, Taiwan; 236 CERN, 1211 Geneva 23, Switzerland

## Abstract

A search for doubly charged Higgs bosons with pairs of prompt, isolated, highly energetic leptons with the same electric charge is presented. The search uses a proton–proton collision data sample at a centre-of-mass energy of 13 TeV corresponding to 36.1 $\text {fb}^{-1}$ of integrated luminosity recorded in 2015 and 2016 by the ATLAS detector at the LHC. This analysis focuses on the decays $H^{\pm \pm }\rightarrow e^{\pm }e^{\pm }$, $H^{\pm \pm }\rightarrow e^{\pm }\mu ^{\pm }$ and $H^{\pm \pm }\rightarrow \mu ^{\pm }\mu ^{\pm }$, fitting the dilepton mass spectra in several exclusive signal regions. No significant evidence of a signal is observed and corresponding limits on the production cross-section and consequently a lower limit on $m(H^{\pm \pm })$ are derived at 95% confidence level. With $\ell ^{\pm }\ell ^{\pm }=e^{\pm }e^{\pm }/\mu ^{\pm }\mu ^{\pm }/e^{\pm }\mu ^{\pm }$, the observed lower limit on the mass of a doubly charged Higgs boson only coupling to left-handed leptons varies from 770 to 870 GeV (850 GeV expected) for $B(H^{\pm \pm }\rightarrow \ell ^{\pm }\ell ^{\pm })=100\%$ and both the expected and observed mass limits are above 450 GeV for $B(H^{\pm \pm }\rightarrow \ell ^{\pm }\ell ^{\pm })=10\%$ and any combination of partial branching ratios.

## Introduction

Events with two prompt, isolated, highly energetic leptons with the same electric charge (same-charge leptons) are produced very rarely in a proton–proton collision according to the predictions of the standard model (SM), but may occur with higher rate in various theories beyond the standard model (BSM). This analysis focuses on BSM theories that contain a doubly charged Higgs particle $H^{\pm \pm }$ using the observed invariant mass of same-charge lepton pairs. In the absence of evidence for a signal, lower limits on the mass of the $H^{\pm \pm }$ particle are set at the $95\%$ confidence level.

Doubly charged Higgs bosons can arise in a large variety of BSM theories, namely in left-right symmetric (LRS) models [1–5], Higgs triplet models [6, 7], the little Higgs model [8], type-II see-saw models [9–13], the Georgi–Machacek model [14], scalar singlet dark matter [15], and the Zee–Babu neutrino mass model [16–18]. Theoretical studies [19–21] indicate that the doubly charged Higgs bosons would be predominantly pair-produced via the Drell–Yan process at the LHC. For this search, the cross-sections utilised to set the final exclusion limits are computed according to the model in Ref. [9].

**Fig. 1:**
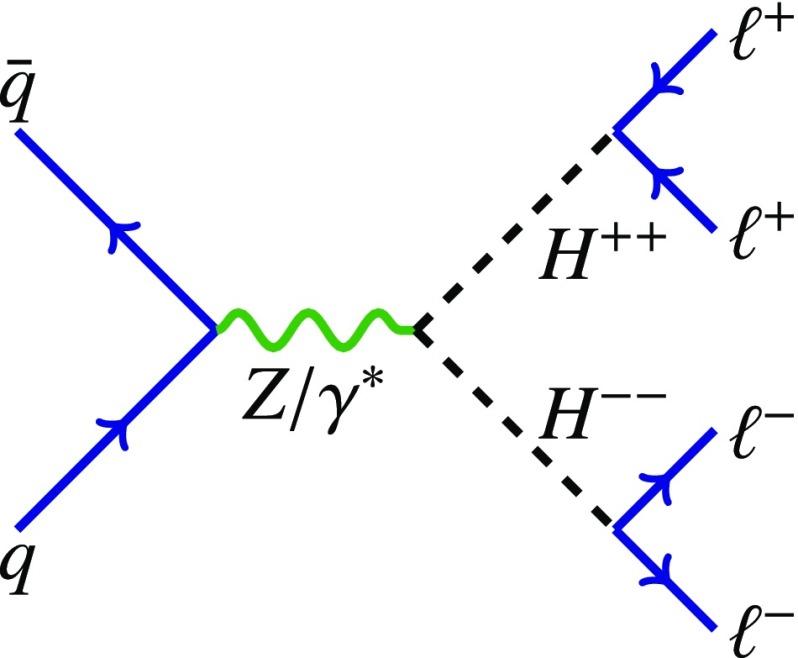
Feynman diagram of the pair production process $pp\rightarrow H^{++}H^{--}$. The analysis studies only the electron and muon channels, where at least one of the lepton pairs is $e^{\pm }e^{\pm }$, $e^{\pm }\mu ^{\pm }$, or $\mu ^{\pm }\mu ^{\pm }$

Doubly charged Higgs particles can couple to either left-handed or right-handed leptons. In LRS models, two cases are distinguished and denoted $H_{L}^{\pm \pm }$ and $H_{R}^{\pm \pm }$. The cross-section for $H_{L}^{++}H_{L}^{--}$ production is about 2.3 times larger than for $H_{R}^{++}H_{R}^{--}$ due to the different couplings to the *Z* boson [22]. Besides the leptonic decay, the $H^{\pm \pm }$ particle can decay into a pair of *W* bosons as well. For low values of the Higgs triplet vacuum expectation value $v_{\Delta }$, it decays almost exclusively to leptons while for high values of $v_{\Delta }$ the decay is mostly to a pair of *W* bosons [9, 12]. In this analysis, the coupling to *W* bosons is assumed to be negligible and only pair production via the Drell–Yan process is considered. The Feynman diagram of the production mechanism is presented in Fig. 1.

The analysis targets only decays of the $H^{\pm \pm }$ particle into electrons and muons, denoted by $\ell$. Other final states *X* that are not directly selected in this analysis are taken into account by reducing the lepton multiplicity of the final state. These states *X* would include, for instance, $\tau$ leptons or *W* bosons, as well as particles which escape detection. The total assumed branching ratio of $H^{\pm \pm }$ is therefore $B(H^{\pm \pm } \rightarrow e^{\pm }e^{\pm })+B(H^{\pm \pm } \rightarrow e^{\pm }\mu ^{\pm }) + B(H^{\pm \pm } \rightarrow \mu ^{\pm }\mu ^{\pm }) + B(H^{\pm \pm } \rightarrow X) = B(H^{\pm \pm } \rightarrow \ell ^{\pm } \ell ^{\pm }) + B(H^{\pm \pm } \rightarrow X) = 100\%$. Moreover, the decay width is assumed to be negligible compared to the detector resolution, which is compatible with theoretical predictions. Two-, three-, and four-lepton signal regions are defined to select the majority of such events. These regions are further divided into unique flavour categories (*e* or $\mu$) to increase the sensitivity. The partial decay width of $H^{\pm \pm }$ to leptons is given by:

$$\begin{aligned} \Gamma (H^{\pm \pm } \rightarrow \ell ^{\pm } \ell '^{\pm }) = k\frac{h^{2}_{\ell \ell '}}{16 \pi } m(H^{\pm \pm }), \end{aligned}$$

with $k=2$ if both leptons have the same flavour ($\ell =\ell '$) and $k=1$ for different flavours. The factor $h_{\ell \ell '}$ has an upper bound that depends on the flavour combination [23, 24]. In this analysis, only prompt decays of the $H^{\pm \pm }$ bosons ($c\tau < {10}\mathrm {\mu }$m) are considered, corresponding to $h_{\ell \ell '} \gtrsim 1.5\times 10^{-6}$ for $m(H^{\pm \pm })=200\,\text {GeV}$. In general, there is no preference for decays into $\tau$ leptons, as the coupling is not proportional to the lepton mass like it is for the SM Higgs boson.

Additional motivation to study cases with $B(H^{\pm \pm } \rightarrow \ell ^{\pm } \ell ^{\pm }) < 100\%$ is given by type-II see-saw models with specific neutrino mass hypotheses resulting in a fixed branching ratio combination [13, 25, 26] which does not necessarily correspond to $B(H^{\pm \pm } \rightarrow \ell ^{\pm } \ell ^{\pm }) = 100\%$.

The ATLAS Collaboration previously analysed data corresponding to 20.3 $\text {fb}^{-1}$ of integrated luminosity which were recorded in 2012 at a centre-of-mass energy of 8 TeV [27]. This study resulted in the most stringent lower limits on the mass of a potential $H^{\pm \pm }_L$ particle. Depending on the flavour of the final-state leptons, the observed limits vary between 465 and 550 GeV assuming $B(H^{\pm \pm }_L\rightarrow \ell ^\pm \ell ^\pm )=100\%$. The analysis presented in this paper extends the one described in Ref. [27] and is based on 36.1 $\text {fb}^{-1}$ of integrated luminosity collected in 2015 and 2016 at a centre-of-mass energy of 13 TeV. A similar search has also been performed by the CMS Collaboration [28].

## ATLAS detector

The ATLAS detector [29] at the LHC is a multi-purpose particle detector with a forward–backward symmetric cylindrical geometry and an almost $4\pi$ coverage in solid angle.^1^ It consists of an inner tracking detector (ID) surrounded by a thin superconducting solenoid providing a 2 T axial magnetic field, electromagnetic (EM) and hadronic calorimeters, and a muon spectrometer. The inner tracking detector covers the pseudorapidity range $|\eta | < 2.5$. It is composed of silicon pixel, silicon micro-strip, and transition radiation tracking detectors. A new innermost layer of pixel detectors [30] was installed prior to the start of data taking in 2015. Lead/liquid-argon (LAr) sampling calorimeters provide electromagnetic energy measurements with high granularity. A hadronic (steel/scintillator-tile) calorimeter covers the central pseudorapidity range ($|\eta | < 1.7$). The end-cap and forward regions are instrumented with LAr calorimeters for both EM and hadronic energy measurements up to $|\eta | = 4.9$. The muon spectrometer surrounds the calorimeters and features three large air-core toroidal superconducting magnets with eight coils each. The field integral of the toroids ranges between 2 to 6 Tm across most of the detector. The muon system includes precision tracking chambers and fast detectors for triggering. A two-level trigger system is used to select events [31] that are interesting for physics analyses. The first-level trigger is implemented as part of the hardware. Subsequently a software-based high-level trigger executes algorithms similar to those used in the offline reconstruction software, reducing the event rate to about 1 kHz.

## Dataset and simulated event samples

The data used in this analysis were collected at centre-of-mass energy of 13 TeV during 2015 and 2016, and correspond to an integrated luminosity of 3.2 ${\text {fb}}^{-1}$ in 2015 and 32.9 ${\text {fb}}^{-1}$ in 2016. The average number of *pp* interactions per bunch crossing in the dataset is 24. Interactions other than the hard-scattering one are referred to as pile-up. The uncertainty on the combined 2015 and 2016 integrated luminosity is $3.2\%$. Following a methodology similar to the one described in Ref. [32], this uncertainty is derived from a preliminary calibration of the luminosity scale using *x*–*y* beam-separation scans performed in August 2015 and May 2016.

Signal candidate events in the electron channel are required to pass a dielectron trigger with a threshold of 17 GeV on the transverse energy ($E_\text {T}$) of each of the electrons. Candidate events in the muon channel are selected using a combination of two single-muon triggers with transverse momentum ($p_{\text {T}}$) thresholds of 26 and 50 GeV. The single-muon trigger with the lower $p_{\text {T}}$ threshold also requires track-based isolation of the muon according to the isolation criteria described in Ref. [33]. Events containing both electrons and muons (mixed channel) are required to pass either the combined electron–muon trigger or any of the triggers used for the muon channel or the electron channel. The combined trigger has an $E_\text {T}$ threshold of 17 GeV for the electron and a $p_\text {T}$ threshold of 14 GeV for the muon. Events with four leptons are selected using a combination of dilepton triggers. In general, single-lepton triggers are more efficient than dilepton triggers. However, single-electron triggers impose stringent electron identification criteria, which interfere with the data-driven background estimation.

An irreducible background originates from SM processes resulting in same-charge leptons, hereafter referred to as prompt background. Prompt background and signal model predictions were obtained from Monte Carlo (MC) simulated event samples which are summarised in Table 1. Prompt background events mainly originate from diboson ($W^\pm W^\pm$ / *ZZ* / *WZ*) and $t\bar{t}X$ processes ($t\bar{t}W$, $t\bar{t}Z$, and $t\bar{t}H$). They also provide a source of reducible background due to charge misidentification in channels that contain electrons.^2^ As described in Sect. 5, the modelling of charge misidentification in simulation deviates from data and consequently charge reconstruction scale factors are derived in a data-driven way and applied to the simulated events to compensate for the differences. The highest-yield process which enters the analysis through charge misidentification is Drell–Yan ($q\bar{q} \rightarrow Z/\gamma ^{*} \rightarrow \ell ^{+}\ell ^{-}$) followed by $t\bar{t}$ production. MC samples are in general normalised using theoretical cross-sections referenced in Table 1. However, yields of some MC samples are considered as free parameters in the likelihood fit, as described in Sect. 7.

**Table 1:** Simulated signal and background event samples: the corresponding event generator, parton shower, cross-section normalisation, PDF set used for the matrix element and set of tuned parameters are shown for each sample. The cross-section in the event generator that produces the sample is used where not specifically stated otherwise

Physics process	Event generator	ME PDF set	Cross-section normalisation	Parton shower	Parton shower tune
Signal					
$H^{\pm \pm }$	Pythia 8.186 [34]	NNPDF2.3NLO [35]	NLO (see Table 2)	Pythia 8.186	A14 [36]
Drell–Yan					
$Z/\gamma ^{*}\rightarrow ee/\tau \tau$	Powheg-Box v2 [37–39]	CT10 [40]	NNLO [41]	Pythia 8.186	AZNLO [42]
Top					
$t\bar{t}$	Powheg-Box v2	NNPDF3.0NLO [43]	NNLO [44]	Pythia 8.186	A14
Single top	Powheg-Box v2	CT10	NLO [45]	Pythia 6.428 [46]	Perugia 2012 [47]
$t\bar{t}W$, $t\bar{t}Z/\gamma ^{*}$	MG5_aMC@NLO2.2.2 [48]	NNPDF2.3NLO	NLO [49]	Pythia 8.186	A14
$t\bar{t}H$	MG5_aMC@NLO2.3.2	NNPDF2.3NLO	NLO [49]	Pythia 8.186	A14
Diboson					
*ZZ*, *WZ*	Sherpa 2.2.1 [50]	NNPDF3.0NLO	NLO	Sherpa	Sherpa default
Other (inc. $W^{\pm }W^{\pm }$)	Sherpa 2.1.1	CT10	NLO	Sherpa	Sherpa default
Diboson Sys.					
*ZZ*, *WZ*	Powheg-Box v2	CT10NLO	NLO	Pythia 8.186	AZNLO

Another source of reducible background arises from events with non-prompt electrons or muons or with other physics objects misidentified as electrons or muons, collectively called ‘fakes’. For both, electrons and muons, this contribution originates within jets, from decays of light-flavour or heavy-flavour hadrons into light leptons. For electrons, a significant component of fakes arises from jets which satisfy the electron reconstruction criteria and from photon conversions. MC samples are not used to estimate this background because the simulation of jets and hadronisation has large uncertainties. Instead, a data-driven approach is used to assess this contribution from production of *W*+jets, $t\bar{t}$ and multi-jet events. The method is validated in specialised validation regions.

The SM Drell–Yan process was modelled using Powheg-Box v2 [37–39] interfaced to Pythia 8.186 [34] for parton showering. The CT10 set of parton distribution functions (PDF) [40] was used to calculate the hard scattering process. A set of tuned parameters called the AZNLO tune [42] was used in combination with the CTEQ6L1 PDF set [51] to model non-perturbative effects. Photos++ version 3.52 [52] was used for photon emissions from electroweak vertices and charged leptons. The generation of the process was divided into 19 samples with subsequent invariant mass intervals to guarantee a good statistical coverage over the entire mass range.

Higher-order corrections were applied to the Drell–Yan simulated events to scale the mass-dependent cross-section computed at next-to-leading order (NLO) in the strong coupling constant with the CT10 PDF set to next-to-next-to-leading order (NNLO) in the strong coupling constant with the CT14NNLO PDF set [41]. The corrections were calculated with VRAP [53] for QCD effects and Mcsanc [54] for electroweak effects. The latter are corrected from leading-order (LO) to NLO.

A sample of $Z\rightarrow ee$ events was generated with Sherpa 2.2.1 [50], in addition to the Powheg prediction, to measure the probability of electron charge misidentification, as explained in Sect. 5. The electron $p_\text {T}$ spectrum is a crucial ingredient for the estimate of this probability and was found to be better described by Sherpa than by Powheg, especially for invariant masses of the electron pair close to the *Z* boson mass. Sherpa uses Comix [55] and OpenLoops [56] to calculate the matrix elements up to two partons at NLO and up to four partons at LO in the strong coupling constant. The merging with the Sherpa parton shower [57] follows the ME+PS@NLO prescription in [58].

The $t\bar{t}$ process was generated with the NLO QCD event generator Powheg-Box v2 which was interfaced to Pythia 8.186 for parton showering. The A14 parameter set [36] was used together with the NNPDF2.3 [35] PDF set for tuning the shower. Furthermore, the PDF set used for generation was NNPDF3.0 [43]. Additionally, top-quark spin correlations were preserved through the use of MadSpin [59]. The predicted $t\bar{t}$ production cross-section is $832^{+20}_{-30}$ (scale) $\pm 35$ (PDF + $\alpha _{\text {S}}$) pb as calculated with Top++2.0 [44] to NNLO in perturbative QCD, including soft-gluon resummation to next-to-next-to-leading-log order. The top-quark mass was assumed to be 172.5 GeV. The scale uncertainty results from independent variations of the factorisation and renormalisation scales, while the second uncertainty is associated with variations of the PDF set and $\alpha _{\text {S}}$, following the PDF4LHC[60] prescription using the MSTW2008 $68\%$ CL NNLO[61], CT10 NNLO [62], and NNPDF2.3 PDF sets.

Single-top-quark events produced in *Wt* final states were generated by Powheg-Box v2 with the CT10 PDF set used in the matrix element calculations. Single-top-quark events in other final states were generated by Powheg-Box v1. This event generator uses the four-flavour scheme for the NLO QCD matrix element calculations together with the fixed four-flavour PDF set CT10f4. The parton shower, hadronisation, and underlying event were simulated with Pythia 6.428 [46] using the CTEQ6L1 PDF set and the corresponding Perugia 2012 tune (P2012) [47]. The top-quark mass was set to 172.5 GeV. The NLO cross-sections used to normalise these MC samples are summarised in Ref. [45].

The $t\bar{t}W$, $t\bar{t}Z$, and $t\bar{t}H$ processes were generated at LO with MadGraph  v2.2.2 [63] and MadGraph  v2.3.2 using the NNPDF2.3 PDF set. Pythia 8.186 was applied for shower modelling configured with the A14 tune [36], as explained in more detail in Ref. [64]. They were normalised using theoretical cross-sections summarised in Ref. [49].

Diboson processes with four charged leptons, three charged leptons and one neutrino, or two charged leptons and two neutrinos were generated with Sherpa 2.2.1, using matrix elements containing all diagrams with four electroweak vertices. They were calculated for up to three partons at LO accuracy and up to one (4$\ell$, 2$\ell$+2$\nu$) or zero partons (3$\ell$+1$\nu$) at NLO QCD using Comix and OpenLoops. The merging with the Sherpa parton shower [57] follows the ME+PS@NLO prescription. The NNPDF3.0NNLO [43] PDF set was used in conjunction with dedicated parton shower tuning by the Sherpa authors.

Diboson processes with one boson decaying hadronically and the other one decaying leptonically were predicted by Sherpa 2.1.1 [50]. They were calculated for up to three additional partons at LO accuracy and up to one (*ZZ*) or zero (*WW*, *WZ*) additional partons at NLO using Comix and OpenLoops matrix element generators. The merging with the Sherpa parton shower [57] follows the ME+PS@NLO prescription. The CT10 PDF set was used in conjunction with a dedicated parton shower tuning. The Sherpa 2.1.1 diboson prediction was scaled by 0.91 to account for differences between the internal electroweak scheme used in this Sherpa version and the $G_\mu$ scheme which is the common default. Similarly, loop-induced diboson production with both gauge bosons decaying fully leptonically was simulated with Sherpa 2.1.1. The prediction is at LO accuracy while up to one additional jet is merged with the matrix element.

Additional diboson samples for *WZ* and *ZZ* production were generated with Powheg-Box v2 to estimate theoretical uncertainties. Pythia 8.186 provided the parton shower. The CT10 PDF set was used for the matrix element calculation while the parton shower was configured with the CTEQL1 PDF set. The non-perturbative effects were modelled using the AZNLO [42] tune.

Signal samples were generated at LO using the LRS package of Pythia 8.186 which implements the $H^{\pm \pm }$ scenario described in Ref. [22]. The program was configured to use the NNPDF23LO PDF set. The $h_{\ell \ell '}$ couplings of lepton pairs were assumed to be the same for $H_R^{\pm \pm }$ and $H_L^{\pm \pm }$ particles. This choice resulted in a good statistical coverage for all possible decay channels. The production of the $H^{\pm \pm }$ was implemented only via the Drell–Yan process. Originally, the cross-section at $\sqrt{s} = 14\,\text {TeV}$ was calculated with NLO accuracy by the authors of Ref. [9]. Subsequently, a rescaling to $\sqrt{s}=13\,\text {TeV}$ with the CTEQ6 PDF [65] set was provided. The cross-sections and corresponding *K*-factors are summarised in Table 2.

Since this analysis exclusively targets the leptonic decays of the $H^{\pm \pm }$ bosons, the vacuum expectation value of the neutral component of the left-handed Higgs triplet ($v^L_{\Delta }$) was set to zero in order to exclude $H^{\pm \pm }\rightarrow WW$ decays. The decay width of the $H^{\pm \pm }$ particle to leptons depends on the $h_{\ell \ell '}$ couplings. These were set to the value $h_{\ell \ell '}=$ 0.02 in all Pythia 8.186 samples. This setting corresponds to a decay width that is negligible compared to the detector resolution. The $h_{\ell \tau }$ and $h_{\tau \tau }$ couplings were fixed at zero. There are 23 MC samples with different $H^{\pm \pm }$ particle masses, starting from 200 GeV up to 1300 GeV in steps of 50 GeV. The ATLAS detector is expected to have the best $H^{\pm \pm }$ mass resolution in the electron–electron final states. Resolutions around 30 GeV for masses of 200–500 GeV and 50–100 GeV for higher masses can be achieved with the event selection defined in Sect. 4. Furthermore, the $H^{\pm \pm }$ mass resolution in electron–muon final states varies from 50 to 150 GeV and from 50 to 200 GeV in muon–muon final states.

For all simulated samples except those obtained with Sherpa, the EvtGen v1.2.0 program [66] was used to model bottom and charm hadron decays. The effect of the pile-up was included by overlaying minimum-bias collisions, simulated with Pythia  8.186, on each generated signal and background event. The number of overlaid collisions is such that the distribution of the average number of interactions per *pp* bunch crossing in the simulation matches the pile-up conditions observed in the data. The pile-up simulation is described in more detail in Ref. [67].

**Table 2:** NLO cross-sections for the pair production of $H_{L}^{++}H_{L}^{--}$ and $H_{R}^{++}H_{R}^{--}$ in *pp* collisions at $\sqrt{s}=13\,\text {TeV}$, together with the correction factors ($K=\sigma _{\mathrm {NLO}}/\sigma _{\mathrm {LO}}$) used to obtain those values from the LO prediction. These *K*-factors are calculated by the authors of Ref. [9] using the CTEQ6 PDF [65]

$m(H^{\pm \pm })\;[\text {GeV}]$	$\sigma (H^{\pm \pm }_{L})\;[\mathrm {fb}]$	*K*-factor $(H^{\pm \pm }_{L})$	$\sigma (H^{\pm \pm }_{R})\;[\mathrm {fb}]$	*K*-factor $(H^{\pm \pm }_{R})$
300	13	1.25	5.6	1.25
350	7.0	1.25	3.0	1.25
400	3.9	1.24	1.7	1.24
450	2.3	1.24	0.99	1.24
500	1.4	1.24	0.61	1.24
600	0.58	1.23	0.25	1.24
700	0.26	1.23	0.11	1.23
800	0.12	1.22	0.054	1.23
900	0.062	1.22	0.027	1.23
1000	0.032	1.22	0.014	1.24
1100	0.017	1.23	0.0076	1.24
1200	0.0094	1.23	0.0042	1.25
1300	0.0052	1.24	0.0023	1.26

The response of the ATLAS detector was simulated using the Geant 4 toolkit [68]. Data and simulated events were reconstructed with the default ATLAS software [69] while simulated events were corrected with calibration factors to better match the performance measured in data.

## Event reconstruction and selection

Events are required to have at least one reconstructed primary vertex with at least two associated tracks with $p_{\text {T}}$ $> 400$ MeV. Among all the vertices in the event the one with the highest sum of squared transverse momenta of the associated tracks is chosen as the primary vertex.

### Event reconstruction

This analysis classifies leptons in two exclusive categories called *tight* and *loose*, defined specifically for each lepton flavour as described below. Leptons selected in the tight category feature a predominant component of prompt leptons, while loose leptons are mostly fakes, which are used for the fake-background estimation. All tracks associated with lepton candidates must have a longitudinal impact parameter with respect to the primary vertex of less than 0.5 mm.

Electron candidates are reconstructed using information from the EM calorimeter and ID by matching an isolated calorimeter energy deposit to an ID track. They are required to have $|\eta |<2.47$, $p_{\text {T}} >30\,\text {GeV}$, and to pass at least the LHLoose identification level based on a multivariate likelihood discriminant [70, 71]. The likelihood discriminant is based on track and calorimeter cluster information. Electron candidates within the transition region between the barrel and endcap electromagnetic calorimeters ($1.37<|\eta |<1.52$) are vetoed due to limitations in their reconstruction quality. The track associated with the electron candidate must have an impact parameter evaluated at the point of closest approach between the track and the beam axis in the transverse plane ($d_0$) that satisfies $|d_0|/\sigma (d_0)<5$, where $\sigma (d_0)$ is the uncertainty on $d_0$. In addition to this, electron candidates are classified as tight if they satisfy the LHMedium working point of the likelihood discriminant and the isolation criteria described in Ref. [70]. This is based on calorimeter cluster and track isolation, which vary to obtain a fixed efficiency for selecting prompt electrons of $99\%$ across $p_{\text {T}}$ and $\eta$. Electrons are classified as loose if they fail to satisfy either of the identification or the isolation criteria.

Muon candidates are selected by combining information from the muon spectrometer and the ID. They satisfy the medium quality criteria described in Ref. [33] and are required to have $p_{\text {T}} >30\,\text {GeV}$, $|\eta |<2.5$ and $|d_0|/\sigma (d_0)<10$. Muon candidates are classified as tight if their impact parameter satisfies $|d_0|/\sigma (d_0)<3.0$ and they satisfy the most stringent isolation working point of the *cut-based* track isolation [70]. Muons are loose if they fail the isolation requirement.

Jets or particles originating from the hadronisation of partons are reconstructed by clustering energy deposits in the calorimeter calibrated at the EM scale. The anti-$k_{t}$ algorithm [72] is used with a radius parameter of 0.4, which is implemented with the FastJet [73] package. The majority of pile-up jets are rejected using the *jet-vertex-tagger* [74], which is a combination of track-based variables providing discrimination against pile-up jets. For all jets the expected average transverse energy contribution from pile-up is subtracted using an area-based $p_{\text {T}}$ density subtraction method and a residual correction derived from the MC simulation, both detailed in Refs. [75, 76]. In this analysis, events containing jets identified as originating from *b*-quarks are vetoed. They are identified with a multivariate discriminant [76] that has a *b*-jet efficiency of $77\%$ in simulated $t\bar{t}$ events and a rejection factor of $\approx 40$ ($\approx 20$) for jets originating from gluons and light quarks (*c*-quarks).

After electron and muon identification, jet calibration, and pile-up jet removal, overlaps between reconstructed particles or jets are resolved. First, electrons are removed if they share a track with a muon. Secondly, ambiguities between electrons and jets are resolved. If a jet is closer than $\sqrt{(\Delta y)^{2} + (\Delta \phi )^{2}} = 0.2$ the jet is rejected. If $0.2< \sqrt{(\Delta y)^{2} + (\Delta \phi )^{2}} < 0.4$ the electron is removed. Finally, if a muon and a jet are closer than $\sqrt{(\Delta y)^{2} + (\Delta \phi )^{2}} = 0.4$, and the jet features less than 3 tracks, the jet is removed. Otherwise the muon is discarded.

### Event selection

In this search, events are classified in independent categories, called *analysis regions*, which serve different purposes. The so-called *control regions* are used to constrain free background parameters in the statistical analysis detailed in Sect. 7. The background model is validated against data in *validation regions*. Both the control and validation regions are designed to reject signal events. A dedicated selection targeting signal events is utilised to define the *signal regions*. The selection criteria utilised for each region are summarised in Table 3. The main variable that defines the type of the region is the invariant mass of same-charge lepton pairs. Invariant masses are required to be above 200 GeV in signal regions and below 200 GeV in most control and validation regions.

The lepton multiplicity in the event is used to define the analysis regions. Events with two or three leptons are required to contain exactly one same-charge lepton pair, while four-lepton events are required to feature two same-charge pairs where the sum of all lepton charges has to be zero. An exception is the *opposite-charge control region* (OCCR) where exactly two electrons with opposite charge are required. In all regions, events with at least one *b*-tagged jet are vetoed, in order to suppress background events arising from top-quark decays. In regions with more than two leptons, events are rejected if any opposite-charge same-flavour lepton pair is within 10 GeV of the *Z* boson mass ($81.2\,\text {GeV}< m(\ell ^{+}\ell ^{-}) < 101.2\,\text {GeV}$). This requirement is applied to reject diboson events featuring a *Z* boson in the final state, and is inverted in *diboson control regions*, where at least one *Z* boson is present. Furthermore, the *Z* boson veto is not applied in four-lepton control and validation regions to increase the available number of simulated diboson events.

The invariant mass of the same-charge lepton pair is used in the final fit of the analysis for the two- and three-lepton regions. In the OCCR, the invariant mass of the opposite-charge lepton pair is used. A lower bound of 60 GeV on the invariant mass is imposed in all regions to discard low-mass events which would potentially bias the background estimation of the analysis while maximising the available number of events.

**Fig. 2:**
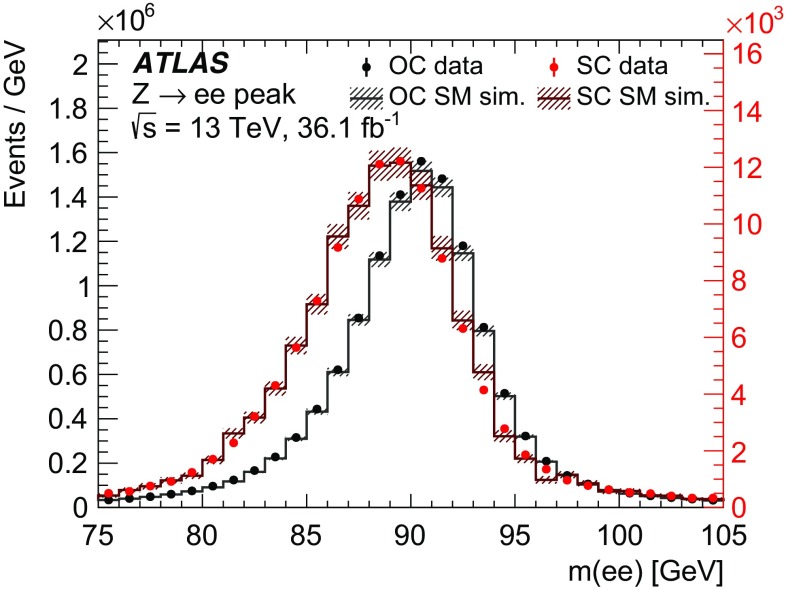
Dielectron mass distributions for opposite-charge (black) and same-charge (red) pairs for data (filled circles) and MC simulation (continuous line). The latter includes a correction for charge misidentification. The hatched band indicates the statistical error and the luminosity uncertainty summed in quadrature applied to MC simulated events

In the electron and mixed channels the lower bound is increased to 90 GeV in the three-lepton regions and to 130 GeV in the two-lepton regions. The motivation for increasing the lower mass bound in regions containing electrons is the data-driven charge misidentification background correction, where the $Z \rightarrow ee$ peak is used to measure the charge misidentification rates (described in Sect. 5). Differences between data and MC simulation in the dielectron same-charge $Z\rightarrow ee$ peak (see Fig. 2) were minimised by construction following the methodology described in Sect. 5, and the $Z\rightarrow ee$ peak was therefore not used in the fit. In the two-lepton regions, this bound is set to 130 GeV to completely remove the *Z* peak region. In the three-lepton regions, where this effect is not as strong, the bound is relaxed to 90 GeV to reduce the statistical uncertainty of the sample. As the charge misidentification background is not present in the muon channel, there is no need to increase the lower mass bound there.

In the mixed channel, events are further divided into two categories, where the same-charge pair features different-flavour leptons or not, indicated by $e^{\pm } \mu ^{\pm } \ell ^{\mp }$ and $e^{\pm } e^{\pm } \mu ^{\mp }$ or $\mu ^{\pm } \mu ^{\pm } e^{\mp }$, respectively.

In order to maximise the sensitivity in two-lepton and three-lepton signal regions (SR1P2L and SR1P3L), additional requirements are imposed on same-charge lepton pairs, regardless of the flavour. These exploit both the boosted decay topology of the $H^{\pm \pm }$ resonance and the high energy of the decay products. The same-charge lepton separation is required to be $\Delta R(\mathrm {\ell ^{\pm },\ell ^{\pm }})<3.5$. Their combined transverse momentum has to be $p_{\text {T}} (\mathrm {\ell ^{\pm }\ell ^{\pm }})>100\,\text {GeV}$.^3^ Finally, the scalar sum of the leptons’ transverse momenta is required to be above 300 GeV in the signal regions. In SR1P2L and SR1P3L, the signal selection efficiency combined with the detector acceptance varies greatly with the assumed branching ratio into light leptons. It is the highest for $B(H^{\pm \pm }\rightarrow \ell ^{\pm } \ell ^{\pm }) \approx 60\%$ where about 40% of signal events are selected either in SR1P2L or SR1P3L. For $B(H^{\pm \pm }\rightarrow \ell ^{\pm } \ell ^{\pm })=100\%$, about 25% of signal events are selected in either of the regions.

In the four-lepton signal region (SR2P4L), the fit variable is the average invariant mass of the two same-charge lepton pairs $\bar{M}\equiv (m^{++}+m^{--})/2$. A selection on the variable $\Delta M/\bar{M} \equiv |m^{++} - m^{--}| / \bar{M}$ is applied to reject background where the two same-charge pairs have inconsistent invariant masses. The $\Delta M/\bar{M}$ requirement is optimised for different flavour combinations which generally feature different mass resolutions. This selection corresponds to $\Delta M$ values which are required to be below 15–50 GeV for $\bar{M}=200\,\text {GeV}$, 30–160 GeV for $\bar{M}=500\,\text {GeV}$, and 50–500 GeV for $\bar{M}=1000\,\text {GeV}$. In the 2P4L signal region, the fraction of signal events that are selected is approximately 50% for the $B(H^{\pm \pm }\rightarrow \ell ^{\pm } \ell ^{\pm })=100\%$ case and lower for branching ratios into light leptons below 100%.

The *same-charge validation region* (SCVR) is used to validate the data-driven fake-background estimation and the charge misidentification effect in the electron channel. The *three-lepton validation region* (3LVR) is used to validate the SM diboson background and fake events with three reconstructed leptons with different proportions across channels. The *four-lepton validation region* (4LVR) is used to validate the diboson modelling in the four-lepton region. Furthermore, the *diboson control region* (DBCR) is used to constrain the diboson background yield in each channel while the opposite-charge control region is used to constrain the Drell–Yan contribution in the electron channel only. The *four-lepton control region* (4LCR) is used to constrain the yield of the diboson background in four-lepton regions.

**Table 3:** Summary of all regions used in the analysis. The table is split into three blocks: the upper block indicates the final states for each region, the middle block indicates the mass range of the corresponding final state, and the lower block indicates the event selection criteria for the region. The application of a selection requirement is indicated by a check-mark (✓). The 2P4L regions include all lepton flavour combinations. In the three lepton regions, $\ell ^{\pm }\ell ^{\pm }\ell '^{\mp }$ indicates that same-charge leptons have the same flavour, while the opposite-sign lepton has a different flavour

Channel	Region								
	Control Regions			Validation Regions			Signal Regions		
	OCCR	DBCR	4LCR	SCVR	3LVR	4LVR	1P2L	1P3L	2P4L
Electron channel	$e^{\pm }e^{\mp }$	$e^{\pm }e^{\pm }e^{\mp }$	$\ell ^{\pm }\ell ^{\pm } \ell ^{\mp }\ell ^{\mp }$	$e^{\pm }e^{\pm }$	$e^{\pm }e^{\pm }e^{\mp }$	$\ell ^{\pm }\ell ^{\pm } \ell ^{\mp }\ell ^{\mp }$	$e^{\pm }e^{\pm }$	$e^{\pm }e^{\pm }e^{\mp }$	$\ell ^{\pm }\ell ^{\pm } \ell ^{\mp }\ell ^{\mp }$
Mixed channel	–	$e^{\pm }\mu ^{\pm }\ell ^{\mp }$		$e^{\pm }\mu ^{\pm }$	$$\begin{array}{c} e^{\pm }\mu ^{\pm }\ell ^{\mp }\\ \ell ^{\pm }\ell ^{\pm }\ell '^{\mp } \end{array}$$		$e^{\pm }\mu ^{\pm }$	$$\begin{array}{c} e^{\pm }\mu ^{\pm }\ell ^{\mp }\\ \ell ^{\pm }\ell ^{\pm }\ell '^{\mp } \end{array}$$	
Muon channel	–	$\mu ^{\pm }\mu ^{\pm }\mu ^{\mp }$		$\mu ^{\pm }\mu ^{\pm }$	$\mu ^{\pm }\mu ^{\pm }\mu ^{\mp }$		$\mu ^{\pm }\mu ^{\pm }$	$\mu ^{\pm }\mu ^{\pm }\mu ^{\mp }$	
$m(e^{\pm }e^{\pm })$ [GeV]	[130, 2000]	[90, 200)	[60, 150)	[130, 200)	[90, 200)	[150, 200)	$[200,\infty )$	$[200,\infty )$	$[200,\infty )$
$m(\ell ^{\pm }\ell ^{\pm })$ [GeV]	–	[90, 200)		[130, 200)	[90, 200)		$[200,\infty )$	$[200,\infty )$	
$m(\mu ^{\pm }\mu ^{\pm })$ [GeV]	–	[60, 200)		[60, 200)	[60, 200)		$[200,\infty )$	$[200,\infty )$	
*b*-jet veto	✓	✓	✓	✓	✓	✓	✓	✓	✓
*Z* veto	–	inverted	–	–	✓	–	–	✓	✓
$\Delta R(\ell ^{\pm },\ell ^{\pm })<3.5$	–	–	–	–	–	–	✓	✓	–
$p_{\mathrm {T}}(\ell ^{\pm }\ell ^{\pm })>100\;\mathrm {GeV}$	–	–	–	–	–	–	✓	✓	–
$\sum |p_{\mathrm {T}}(\ell )|>300\;\mathrm {GeV}$	–	–	–	–	–	–	✓	✓	–
$\Delta M/\bar{M}$ requirement	–	–	–	–	–	–	–	–	✓

## Background composition and estimation

Prompt SM backgrounds in all regions are estimated using the simulated samples listed in Sect. 3. Prompt light leptons are defined as leptons originating from *Z*, *W*, and *H* boson decays or leptons from $\tau$ decays if the $\tau$ has a prompt source (e.g. $Z\rightarrow \tau \tau$). MC events containing at least one non-prompt or fake selected tight or loose lepton are discarded to avoid an overlap with the data-driven fake-background estimation. Prompt electrons in the remaining simulated events are corrected to account for different charge misidentification probabilities in data and simulation.

Electron charge misidentification is caused predominantly by bremsstrahlung. The emitted photon can either convert to an electron–positron pair, which happens in most of the cases, or traverse the inner detector without creating any track. In the first case, the cluster corresponding to the initial electron can be matched to the wrong-charge track, or most of the energy is transferred from one track to the other because of the photon. In case of photon emission without subsequent pair production, the electron track has usually very few hits only in the silicon pixel layers, and thus a short lever arm on its curvature. Because the electron charge is derived from the track curvature, it could be incorrectly determined while the electron energy is likely appropriate as the emitted photon deposits all of its energy in the EM calorimeter as well. For a similar reason high-energy electrons are more often affected by charge misidentification, as their tracks are approximately straight and therefore challenging for the curvature measurement. The modelling of charge misidentification in simulation deviates from data due to the complex processes involved, which particularly rely on a very precise description of the detector material. A correction is obtained by comparing the charge misidentification probability measured in data to the one in simulation. The charge misidentification probability is extracted by performing a likelihood fit on a dedicated $Z \rightarrow ee$ data sample (see Fig. 2). Electron pairs are selected around the *Z* boson peak and categorised in opposite-charge (OC) and same-charge (SC) selections with the invariant mass requirements $|m_{\text {OC}}(ee)-m(Z)|<14\;\hbox {GeV}$ and $|m_{\text {SC}}(ee)-m(Z)|<15.8\;\hbox {GeV}$, respectively. Events from contributions other than $Z \rightarrow ee$ are subtracted from the peak regions. They are modelled with simulation and their normalisation is determined from data in mass windows around the *Z* peak defined as $14\;\hbox {GeV}\;< |m_{\text {OC}}(ee)-m(Z)| < 18\;\hbox {GeV}\;$ for OC and $15.8\;\hbox {GeV}\;< |m_{\text {OC}}(ee)-m(Z)| < 31.6\;\hbox {GeV}\;$ for SC. The number of OS and SC electron pairs in the two regions ($N^{ij}=N^{ij}_{\text {SC}} + N^{ij}_{\text {OC}}$) are then used as inputs of the likelihood fit.

The probability to observe $N^{ij}_{\text {SC}}$ same-charge pairs is the Poisson probability:

$$\begin{aligned} f(N^{ij}_{\text {SC}};\lambda )=\frac{\lambda ^{N^{ij}_{\text {SC}}} \mathrm {e}^{-\lambda }}{N^{ij}_{\text {SC}}!}, \end{aligned}$$

with $\lambda = N^{ij}(P_i(1-P_j) + P_j(1-P_i))$ denoting the expected number of same-charge pairs in bin (*i*, *j*), where *i* and *j* indicate the kinematic configuration of the two electrons in the pair, given the charge misidentification probabilities $P_i$ and $P_j$. $N^{ij}_{\text {SC}}$ is the measured number of same-charge pairs. The formula for the negative log likelihood used in the likelihood fit is given in Eq. 1:

1 $$\begin{aligned} -\log L(\varvec{P}|\varvec{N_{\text {SC}}},\varvec{N}) = \sum \limits _{i,j}\log (N^{ij}(P_i(1-P_j) + P_j(1-P_i)))N^{ij}_{\text {SC}}-N^{ij}(P_i(1-P_j) + P_j(1-P_i)). \end{aligned}$$

**Fig. 3:**
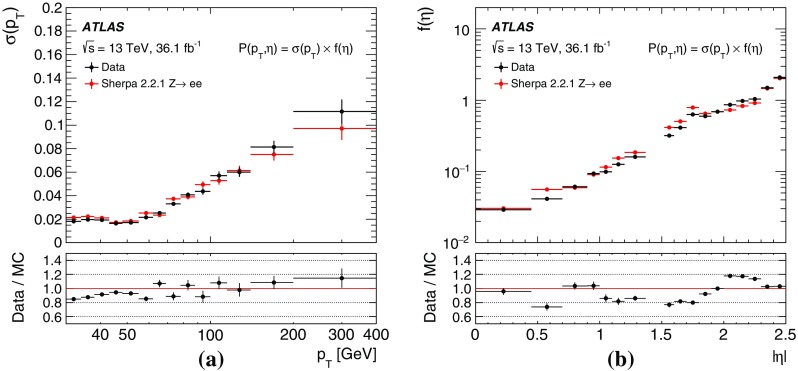
Comparison of the factors composing the charge misidentification probability $P(p_{\text {T}},\eta )=\sigma (p_{\text {T}}) \times f(\eta )$ measured in data and in simulation using the likelihood fit in the $Z/\gamma ^* \rightarrow ee$ region. The area of the distribution describing $f(\eta )$ was set to unity (see text for details). Error bars correspond to the statistical uncertainties estimated with the likelihood fit. Plot (**a**) shows the charge misidentification probability component as a function of $p_{\text {T}}$ and plot (**b**) shows the component as a function of $|\eta |$

The charge misidentification probability is parameterised as a function of electron $p_{\text {T}}$ and $\eta$, $P(p_{\text {T}},\eta )=\sigma (p_{\text {T}}) \times f(\eta )$. The binned values, $\sigma (p_{\text {T}})$ and $f(\eta )$, are free parameters in the likelihood fit. To ensure the proper normalisation of $P(p_{\text {T}},\eta )$, the area of the distribution describing $f(\eta )$ was set to unity. The charge misidentification probability is measured with the same method in a simulated $Z/\gamma ^* \rightarrow ee$ sample and in data. The comparison of the result is shown in Fig. 3. All prompt electrons in simulated events are corrected with charge reconstruction scale factors. The scale factors are defined as $P(p_{\text {T}},\eta ;\text {data})/P(p_{\text {T}},\eta ;\text {MC}))$ if the charge is wrongly reconstructed and $\left( 1-P(p_{\text {T}},\eta ;\text {data})\right) /\left( 1-P(p_{\text {T}},\eta ;\text {MC}\right)$ if the charge is properly reconstructed.

The fake-lepton background is estimated with a data-driven approach, the so-called ‘fake factor’ method, as described in Ref. [27]. The *b*-jet veto significantly reduces fake leptons from heavy-flavour decays. Most of the fake leptons still passing the analysis selection originate from in-flight decays of mesons inside jets, jets misreconstructed as electrons, and conversions of initial- and final-state radiation photons. The fake factor method provides an estimation of events with fake leptons in analysis regions by extrapolating the yields from the so-called ‘side-band regions’. For each analysis region a corresponding side-band region is defined. It requires exactly the same selection and lepton multiplicity except that at least one lepton must fail to satisfy the tight identification criteria. The ratio of tight to loose leptons is measured in dedicated ‘fake-enriched regions’. It is determined as a function of lepton flavour, $p_{\text {T}}$, and $\eta$, and referred to as the ‘fake factor’ ($F(p_{\text {T}},\eta ,\text {flavour})$). It describes the probability for a fake lepton to be identified as a tight lepton. The definitions of the fake-enriched regions for the electron and muon channels are reported in Table 4. In the measurement of the fake factor, a requirement on the unbalanced momentum in the transverse plane of the event, $E^{\mathrm {miss}}_{\mathrm {T}}$, is imposed to reject $W+{\mathrm {jets}}$ events and to further enrich the regions with fake leptons. The fake factor method relies on the assumption that no prompt leptons appear in the fake-enriched samples. This assumption is not fully correct with the imposed selection. Therefore, the number of residual prompt leptons in the fake-enriched regions is estimated using simulation and subtracted from the numbers of tight and loose leptons used to measure the fake factors.

The number of events in the analysis regions containing at least one fake lepton, $N^{\mathrm {fake}}$, is estimated from the side-bands. Data are weighted with fake factors according to the loose lepton multiplicity of the region:

$$\begin{aligned} N^{\mathrm {fake}} = \sum _{i=1}^{N^{\mathrm {data}}_{\mathrm {SB}} }(-1)^{N_{L,i}+1}\prod _{l=1}^{N_{L,i}}F_{l} \; - \; \sum _{i=1}^{N^{\mathrm {MC}}_{\mathrm {SB}} }(-1)^{N_{L,i}+1}\prod _{l=1}^{N_{L,i}}F_{l}, \end{aligned}$$

with $N^{\mathrm {data}}_{\mathrm {SB}}$ denoting the number of data events in the side-band, $N_{L,i}$ is the loose lepton multiplicity in the *i*-th event of the side-band region and *l* indicates the loose lepton. The contamination of prompt leptons in the side-band region is subtracted using simulated events, denoted by $N^{\mathrm {MC}}_{\mathrm {SB}}$.

Dedicated two-lepton and three-lepton validation regions, defined in Table 3, are used to verify the data-driven fake-lepton estimation in regions as similar to the signal regions as possible. They are designed to contain only a negligible number of signal events. Orthogonality between signal and validation regions is ensured by requiring the invariant mass of the same-charge lepton pair $m(\ell ^{\pm }\ell ^{\pm })$ to be less than 200 GeV in the validation regions. Furthermore, diboson modelling and the electron charge misidentification backgrounds are tested. Each background estimation is validated in the corresponding regions, defined to be enriched in the given contribution.

**Table 4:** Selection criteria defining the fake-enriched regions used to measure the ratio of the numbers of tight and loose leptons, the so-called fake factor, for the electron and muon channels

Selection for fake-enriched regions	
Muon channel	Electron channel
Single-muon trigger	Single-electron trigger
*b*-jet veto	*b*-jet veto
One muon and one jet	One electron
$p_{\mathrm {T}}(\mathrm {jet})>35\;\mathrm {GeV}$	Number of tight electrons < 2
$\Delta \phi (\mu ,\mathrm {jet})>2.7$	$m(ee)\notin [71.2,111.2]\;\mathrm {GeV}$
$E_{\mathrm {T}}^{\mathrm {miss}}<40\;\mathrm {GeV}$	$E_{\mathrm {T}}^{\mathrm {miss}}<25\;\mathrm {GeV}$

**Fig. 4:**
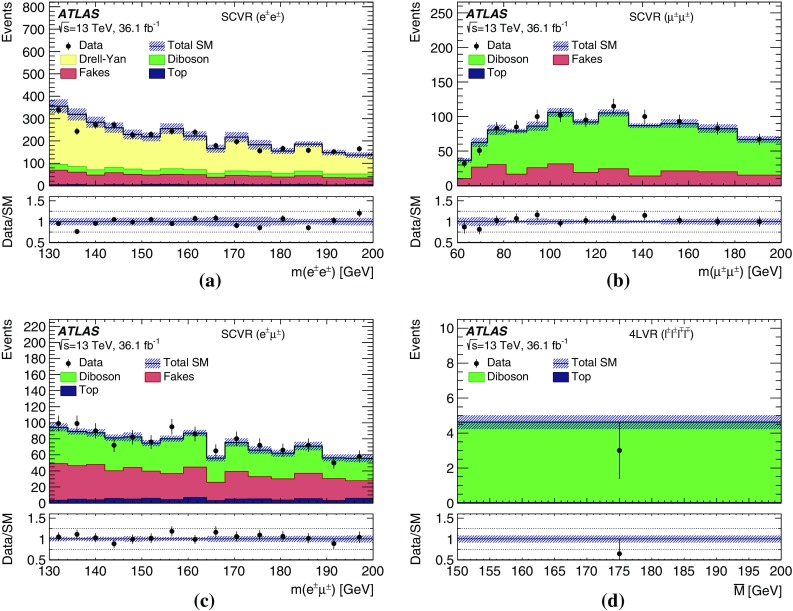
Distributions of dilepton mass for data and SM background predictions in two- and four-lepton validation regions: **a** the electron–electron, **b** the muon–muon, and **c** the electron–muon two-lepton validation regions, as well as **c** the four-lepton validation region. The hatched bands include all systematic uncertainties post-fit, with the correlations between various sources taken into account

**Fig. 5:**
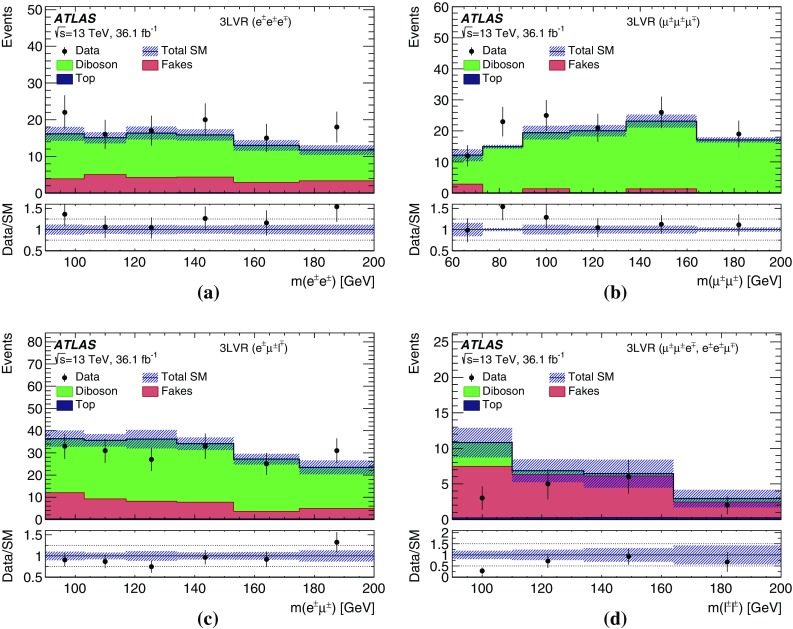
Distribution of dilepton mass for data and SM background predictions in three-lepton validation regions: **a** the three-electron validation region, **b** the three-muon validation region, **c** the 3LVR with an electron–muon same-charge pair ($e^{\pm }\mu ^{\pm }\ell ^{\mp }$), and **d** the 3LVR with a same-flavour same-charge pair ($e^{\pm }e^{\pm }\mu ^{\mp }$ or $\mu ^{\pm }\mu ^{\pm }e^{\mp }$). The hatched bands include all systematic uncertainties post-fit, with the correlations between various sources taken into account

Figures 4 and 5 present all validation regions sensitive to different background sources: same-charge two-lepton validation regions (SCVR) for testing the charge misidentification background modelling and fake-background predictions, and three-lepton and four-lepton validation regions (3LVR and 4LVR) for testing the diboson modelling. Good background modelling is observed in all these regions.

## Systematic uncertainties

Several sources of systematic uncertainty are accounted for in the analysis. These correspond to experimental and theoretical sources affecting both background and signal predictions. All considered sources of systematic uncertainty affect the total event yield, and all except the uncertainties on the luminosity and cross section also affect the distributions of the variables used in the fit (Sect. 7).

The cross-sections used to normalise the simulated samples are varied to account for the scale and PDF uncertainties in the cross-section calculation. The variation is $6\%$ for diboson production [77], $13\%$ for $t\bar{t}W$ production, $12\%$ for $t\bar{t}Z$ production, and $8\%$ for $t\bar{t}H$ production [49]. The theoretical uncertainty in the Drell–Yan background is estimated by PDF eigenvector variations of the nominal PDF set, variations of PDF scale, $\alpha _{\text {S}}$, electroweak corrections, and photon-induced corrections. The effect of the PDF choice is considered by comparing the nominal PDF set to several others, namely CT10NNLO [62], MMHT14 [78], NNPDF3.0 [43], ABM12 [79], HERAPDF2.0 [80, 81], and JR14 [82]. An envelope is constructed by taking into account the largest deviations from the nominal choice. The predominant prompt background, arising from diboson production, is assigned an additional theoretical uncertainty by comparing the nominal Sherpa 2.2.1 prediction with the Powheg prediction. This uncertainty varies from 5 to 10%. Furthermore, the theoretical uncertainty in the NLO cross-section for $pp\rightarrow H^{++}H^{--}$ is reported to be about 15% [9]. It includes the renormalization and factorization scale dependence and the uncertainty in the parton densities. Lastly, the theoretical uncertainty in the simulated $pp\rightarrow H^{++}H^{--}$ events is assessed by varying the A14 parameter set in Pythia 8.186 and choosing alternative PDFs CTEQ6L1 and CT09MC1 [83]. The impact on the signal acceptance is found to be negligible.

A significant contribution arises from the statistical uncertainty in the MC samples and data sideband regions. Analysis regions have a very restrictive selection and only a small fraction of the initially generated MC events remains after applying all requirements. The statistical uncertainty varies from 5 to 40% depending on the signal region.

Experimental systematic uncertainties due to different reconstruction, identification, isolation, and trigger efficiencies of leptons in data compared to simulation are estimated by varying the corresponding scale-factors. They are at most 3% and less significant than the other systematic uncertainties and MC statistical uncertainties. The same is true for lepton energy or momentum calibration.

The experimental uncertainty related to the charge misidentification probability of electrons arises from the statistical uncertainty of both the data and the simulated sample of $Z/\gamma ^* \rightarrow ee$ events used to measure this probability. The uncertainty ranges between 10 and $20\%$ as a function of the electron $p_{\text {T}}$ and $\eta$. Possible systematic effects were investigated by altering the selection requirements imposed on the invariant mass used to select $Z/\gamma ^* \rightarrow ee$ events analysed to measure the misidentification probability. The effects estimated with this method are found to be negligible compared to the statistical uncertainty.

The experimental systematic uncertainty in the data-driven estimate of the fake-lepton background is evaluated by varying the nominal fake factor to account for different effects. The $E^{\mathrm {miss}}_{\mathrm {T}}$ requirement is altered to consider variations in the $W+\mathrm {jets}$ composition. The flavour composition of the fakes is investigated by requiring an additional recoiling jet in the electron channel and changing the definition of the recoiling jet in the muon channel. Furthermore, the transverse impact parameter criterion for tight muons (defined in Sect. 4.1) is varied by one standard deviation. Finally, in the fake-enriched regions, the normalisation of the subtracted simulated samples, to remove the prompt lepton component, is altered within its uncertainties. This accounts for uncertainties related to the luminosity, the cross-section, and the corrections applied to simulation-based predictions. The statistical uncertainty in the fake factors is added in quadrature to the total systematic error. The uncertainty ranges between $10\%$ and $20\%$ across all $p_{\text {T}}$ and $\eta$ bins.

**Fig. 6:**
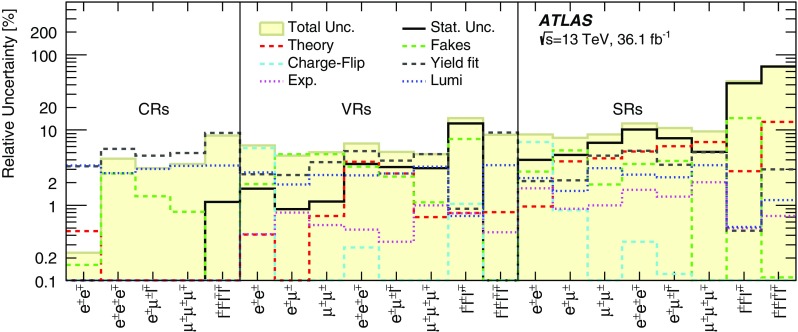
Relative uncertainties in the total background yield estimation after the fit. ‘Stat. Unc.’ corresponds to reducible and irreducible background statistical uncertainties. ‘Yield fit’ corresponds to the uncertainty arising from fitting the yield of diboson and Drell–Yan backgrounds. ‘Lumi’ corresponds to the uncertainty in the luminosity. ‘Theory’ indicates the theoretical uncertainty in the physics model used for simulation (e.g. cross-sections). ‘Exp.’ indicates the uncertainty in the simulation of electron and muon efficiencies (e.g. trigger, identification). ‘Fakes’ is the uncertainty associated with the model of the fake background. Individual uncertainties can be correlated, and do not necessarily add in quadrature to the total background uncertainty, which is indicated by ‘Total Unc.’

The total relative systematic uncertainty after the fit (Sect. 7), and its breakdown into components, is presented in Fig. 6. All experimental systematic uncertainties discussed here affect the signal samples as well as the background.

## Statistical analysis and results

The statistical analysis package HistFitter [84] was used to implement a maximum-likelihood fit of the dilepton invariant mass distribution in all control and signal regions, and the $\bar{M}$ distribution in four-lepton regions to obtain the numbers of signal and background events. The likelihood is the product of a Poisson probability density function describing the observed number of events and Gaussian distributions to constrain the nuisance parameters associated with the systematic uncertainties. The widths of the Gaussian distributions correspond to the magnitudes of these uncertainties, whereas Poisson distributions are used for MC simulation statistical uncertainties. Furthermore, additional free parameters are introduced for the Drell–Yan and the diboson background contributions, to fit their yields in the analysis regions. Fitting the yields of the largest backgrounds reduces the systematic uncertainty in the predicted yield from SM sources. The fitted normalisations are compatible with their SM predictions within the uncertainties. The diboson yield is described by four free parameters, each corresponding to a different diboson region: electron channel, muon channel, mixed channel, and the four-lepton channel. After the fit, the compatibility between the data and the expected background was assessed. For various branching ratio assumptions, $95\%$ CL upper limits were set on the $pp\rightarrow H^{++}H^{--}$ cross-section using the $\text {CL}_{\text {s}}$ method [85].

### Fit results

**Fig. 7:**
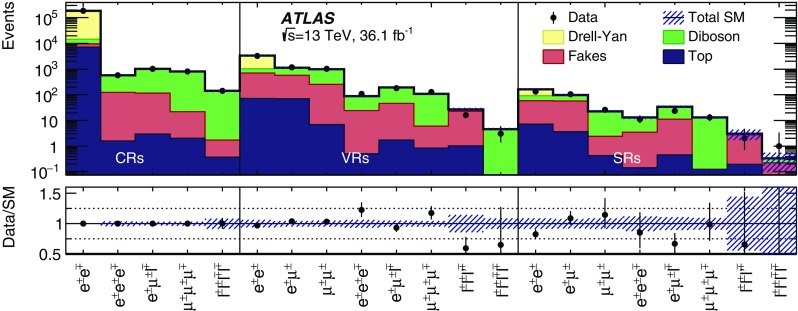
Number of observed and expected events in the control, validation, and signal regions for all channels considered. The background expectation is the result of the fit described in the text. The hatched bands include all systematic uncertainties post-fit with the correlations between various sources taken into account. The notation $\ell ^{\pm }\ell '^{\pm }\ell ^{\mp }$ indicates that the same-charge leptons have different flavours and $\ell ^{\pm }\ell ^{\pm }\ell '^{\mp }$ indicates that same-charge leptons have the same flavour, while the opposite-charge lepton has a different flavour

**Fig. 8:**
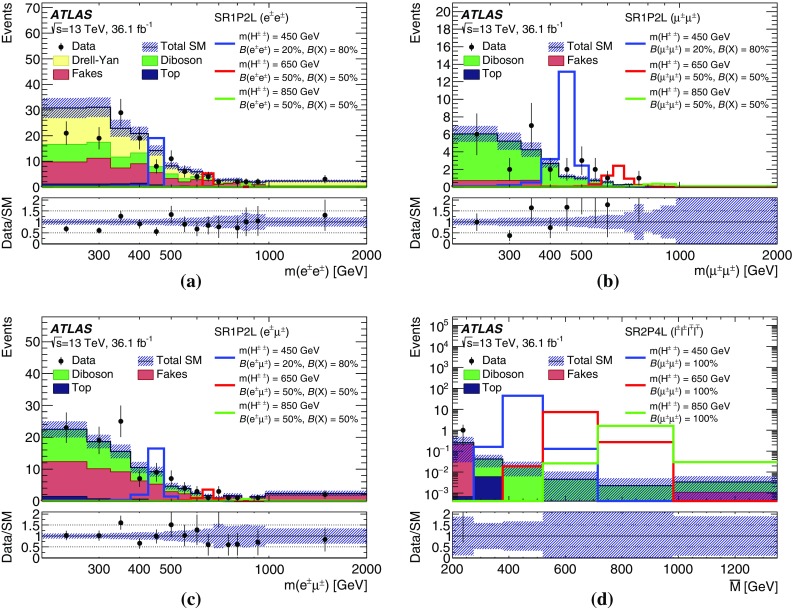
Distributions of $m(\ell ^{\pm }\ell ^{\pm })$ in representative signal regions, namely **a** the electron–electron two-lepton signal region (SR1P2L), **b** the muon–muon two-lepton signal region (SR1P2L), **c** the electron–muon two-lepton signal region (SR1P2L), and **d** the four-lepton signal region (SR2P4L). The hatched bands include all systematic uncertainties post-fit with the correlations between various sources taken into account. The solid coloured lines correspond to signal samples, normalised using the theory cross-section, with the $H^{\pm \pm }$ mass and decay modes marked in the legend

**Fig. 9:**
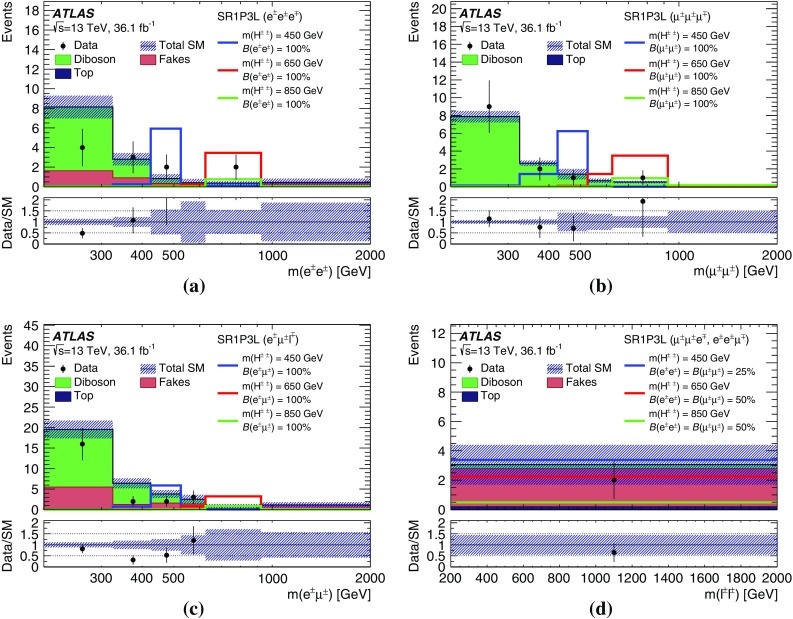
Distributions of $m(\ell ^{\pm }\ell ^{\pm })$ in three-lepton signal regions, namely **a** the three-electron SR (SR1P3L), (b) the three-muon SR (SR1P3L), (**c**) the SR1P3L with an electron–muon same-charge pair ($e^{\pm }\mu ^{\pm }\ell ^{\mp }$), and (d) the SR1P3L with a same-flavour same-charge pair ($e^{\pm }e^{\pm }\mu ^{\mp }$ or $\mu ^{\pm }\mu ^{\pm }e^{\mp }$). The hatched bands include all systematic uncertainties post-fit with the correlations between various sources taken into account. The solid coloured lines correspond to signal samples, normalised using the theory cross-section, with the $H^{\pm \pm }$ mass and decay modes marked in the legend

The observed and expected yields in all control, validation, and signal regions used in the analysis are presented in Fig. 7 and summarised in Tables 5, 6, 7. No significant excess is observed in any of the signal regions. Correlations between various sources of uncertainty are evaluated and used to estimate the total uncertainty in the SM background prediction. Two- and four-lepton signal regions are presented in Fig. 8 and three-lepton signal regions are presented in Fig. 9. In the four-lepton signal region only one data event is observed. It is an $e^{+}\mu ^{+}e^{-}\mu ^{-}$ event with invariant masses of 228 and 207 GeV for the same-charge lepton pairs.

**Table 5:** The number of predicted background events in control regions after the fit, compared to the data. Uncertainties correspond to the total uncertainties in the predicted event yields, and are smaller for the total than the sum of the components in quadrature due to correlations between these components. Due to rounding the totals can differ from the sums of components. Background processes with a negligible yield are marked with the en dash (–)

	OCCR	DBCR	DBCR	DBCR	4LCR
	$e^{\pm }e^{\mp }$	$e^{\pm }e^{\pm }e^{\mp }$	$e^{\pm }\mu ^{\pm }\ell ^{\mp }$	$\mu ^{\pm }\mu ^{\pm }\mu ^{\mp }$	$\ell ^{\pm }\ell ^{\pm }\ell ^{\mp }\ell ^{\mp }$
Observed events	184,569	576	1025	797	140
Total background	184,570±430	574±24	1025±32	797±28	140±12
Drell–Yan	169,980±990	–	–	–	–
Diboson	5060±900	449±28	909±35	775±29	138±12
Fakes	2340±300	123±15	113±14	19.9±6.5	1.31±0.16
Top	7200±250	1.58±0.06	2.90±0.11	2.04±0.08	0.37±0.01

**Table 6:** The number of predicted background events in two-lepton and four-lepton validation regions (top) and three-lepton validation regions (bottom) after the fit, compared to the data. Uncertainties correspond to the total uncertainties in the predicted event yields, and are smaller for the total than the sum of the components in quadrature due to correlations between these components. Due to rounding the totals can differ from the sums of components. Background processes with a negligible yield are marked with the en dash (–)

	SCVR	SCVR	SCVR	4LVR
	$e^{\pm }e^{\pm }$	$e^{\pm }\mu ^{\pm }$	$\mu ^{\pm }\mu ^{\pm }$	$\ell ^{\pm }\ell ^{\pm }\ell ^{\mp }\ell ^{\mp }$
Observed events	3237	1162	1006	3
Total background	3330±210	1119±51	975±50	4.62±0.40
Drell–Yan	2300±190	–	–	–
Diboson	319±25	547±23	719±30	4.59±0.4
Fakes	640±65	502±54	249±47	–
Top	71.5±6.8	70.5±2.6	6.93±0.27	0.033±0.001

	3LVR	3LVR	3LVR	3LVR
	$e^{\pm }e^{\pm }e^{\mp }$	$e^{\pm }\mu ^{\pm }\ell ^{\mp }$	$\mu ^{\pm }\mu ^{\pm }\mu ^{\mp }$	$\mu ^{\pm }\mu ^{\pm }e^{\mp }, e^{\pm }e^{\pm }\mu ^{\mp }$
Observed events	108	180	126	16
Total background	88.1±5.8	192.9±9.9	107.0±5.1	27.0±3.9
Diboson	64.4±5.8	147.3±9.0	100.9±5.0	4.72±0.79
Fakes	23.3±3.0	43.9±4.9	5.3±1.2	21.3±3.4
Top	0.50±0.03	1.73±0.09	0.82±0.05	1.01±0.15

**Table 7:** The number of predicted background events in two-lepton and four-lepton signal regions (top) and three-lepton signal regions (bottom) after the fit, compared to the data. Uncertainties correspond to the total uncertainties in the predicted event yields, and are smaller for the total than the sum of the components in quadrature due to correlations between these components. Due to rounding the totals can differ from the sums of components. Background processes with a negligible yield are marked with the en dash (–)

	SR1P2L	SR1P2L	SR1P2L	SR2P4L
	$e^{\pm }e^{\pm }$	$e^{\pm }\mu ^{\pm }$	$\mu ^{\pm }\mu ^{\pm }$	$\ell ^{\pm }\ell ^{\pm }\ell ^{\mp }\ell ^{\mp }$
Observed events	132	106	26	1
Total background	160${\pm }$14	97.1${\pm }$7.7	22.6${\pm }$2.0	0.33${\pm }$0.23
Drell–Yan	70${\pm }$10	–	–	–
Diboson	30.5${\pm }$3.0	40.4${\pm }$4.5	20.3${\pm }$1.8	0.11${\pm }$0.06
Fakes	52.2${\pm }$5.0	53.1${\pm }$5.8	1.94${\pm }$0.47	0.22${\pm }$0.19
Top	7.20${\pm }$0.97	3.62${\pm }$0.53	0.42${\pm }$0.03	0.007${\pm }$0.002

	SR1P3L	SR1P3L	SR1P3L	SR1P3L
	$e^{\pm }e^{\pm }e^{\mp }$	$e^{\pm }\mu ^{\pm }\ell ^{\mp }$	$\mu ^{\pm }\mu ^{\pm }\mu ^{\mp }$	$\mu ^{\pm }\mu ^{\pm }e^{\mp }, e^{\pm }e^{\pm }\mu ^{\mp }$
Observed events	11	23	13	2
Total background	13.0${\pm }$1.6	34.2${\pm }$3.6	13.2${\pm }$1.3	3.1${\pm }$1.4
Diboson	9.5${\pm }$1.3	23.1${\pm }$2.9	13.1${\pm }$1.3	0.27${\pm }$0.14
Fakes	3.3${\pm }$0.67	10.7${\pm }$1.7	–	2.6${\pm }$1.2
Top	0.14${\pm }$0.02	0.45${\pm }$0.04	0.12${\pm }$0.01	0.19${\pm }$0.08

**Fig. 10:**
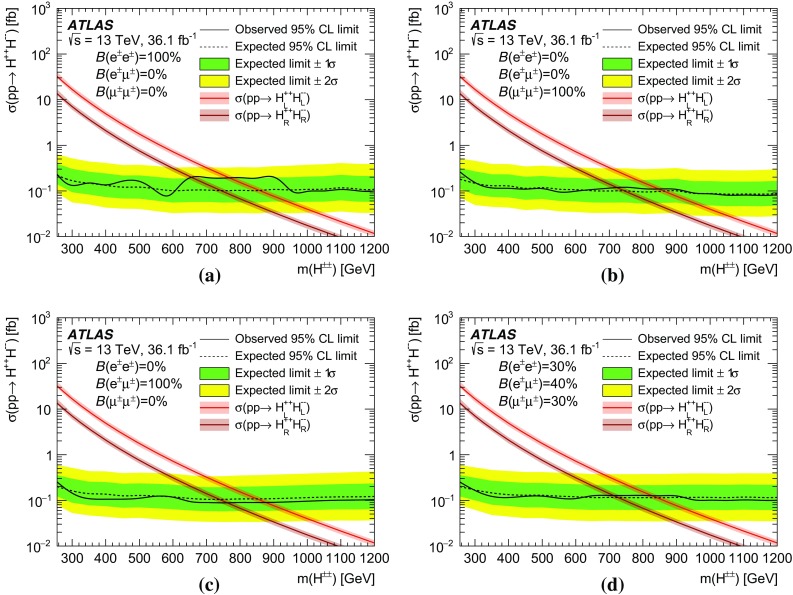
Upper limit on the cross-section for $pp\rightarrow H^{++}H^{--}$ for several branching ratio values presented in the form $B(ee)/B(e\mu )/B(\mu \mu )$: **a** $100\%/0\%/0\%$, **b** $0\%/0\%/100\%$, **c** $0\%/100\%/0\%$, and **d** $30\%/40\%/30\%$. The theoretical uncertainty in the cross-section for $pp\rightarrow H^{++}H^{--}$ is presented with the shaded band around the central value

The likelihood fit to the two-, three-, and four-lepton control and signal regions was designed to fully exploit the pair production of the $H^{\pm \pm }$ boson with its boosted topology and lepton multiplicity. For $B(H^{\pm \pm }\rightarrow \ell ^{\pm }\ell ^{\pm })=100\%$ the production cross-section is excluded down to 0.1 fb, corresponding to 3–4 signal events, which is the theoretical limit of a $95\%$ CL exclusion. Some representative cross-section upper limits as a function of the $H^{\pm \pm }$ boson mass are presented in Fig. 10, for different combinations of the branching ratios for decay into light-lepton pairs.

**Fig. 11:**
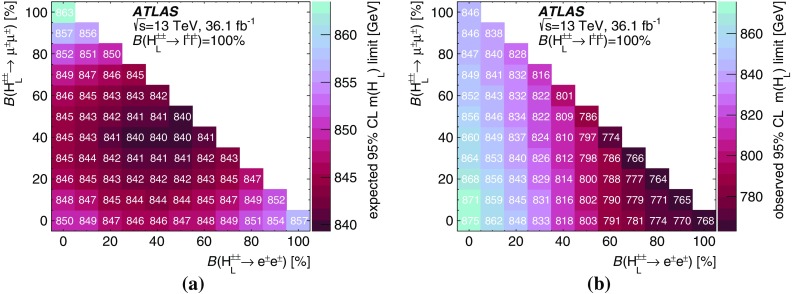
The **a** expected and **b** observed lower limits on the $H_{L}^{\pm \pm }$ boson mass for all branching ratio combinations that sum to $100\%$

**Fig. 12:**
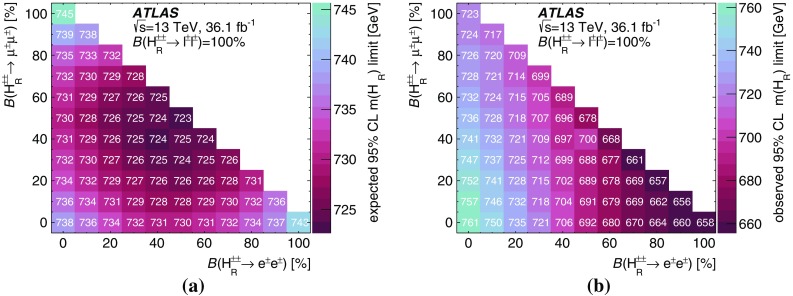
The **a** expected and **b** observed lower limits on the $H_{R}^{\pm \pm }$ boson mass for all branching ratio combinations that sum to $100\%$

**Fig. 13:**
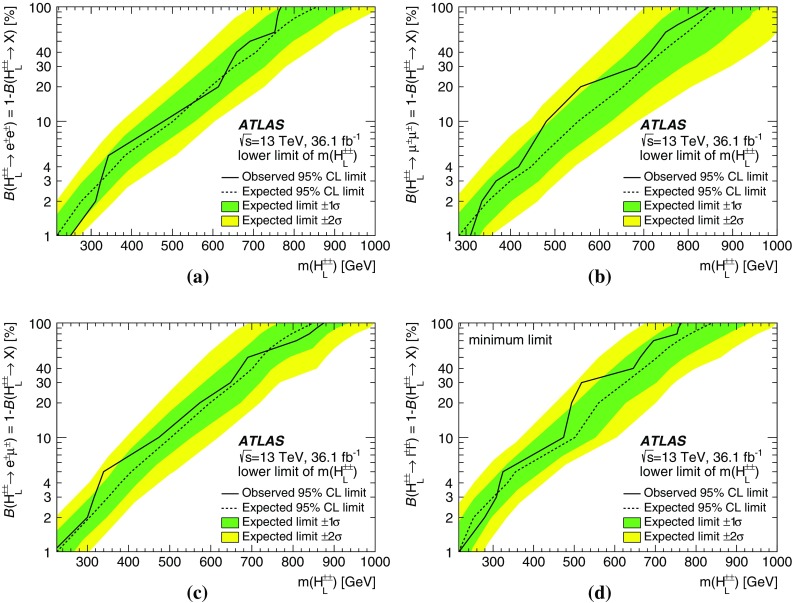
Lower limit on the $H_{L}^{\pm \pm }$ boson mass as a function of the branching ratio $B(H_{L}^{\pm \pm }\rightarrow \ell ^{\pm }\ell ^{\pm })$. Several cases are presented: **a** $H_{L}^{\pm \pm }$ decays only into electrons and “*X*”, **b** $H_{L}^{\pm \pm }$ decays only into muons and “*X*”, and **c** $H_{L}^{\pm \pm }$ decays only into electron–muon pairs and “*X*”, with “*X*” not entering any of the signal regions. Plot **d** shows the minimum observed and expected limit as a function of $B(H_{L}^{\pm \pm }\rightarrow \ell ^{\pm }\ell ^{\pm })$

**Fig. 14:**
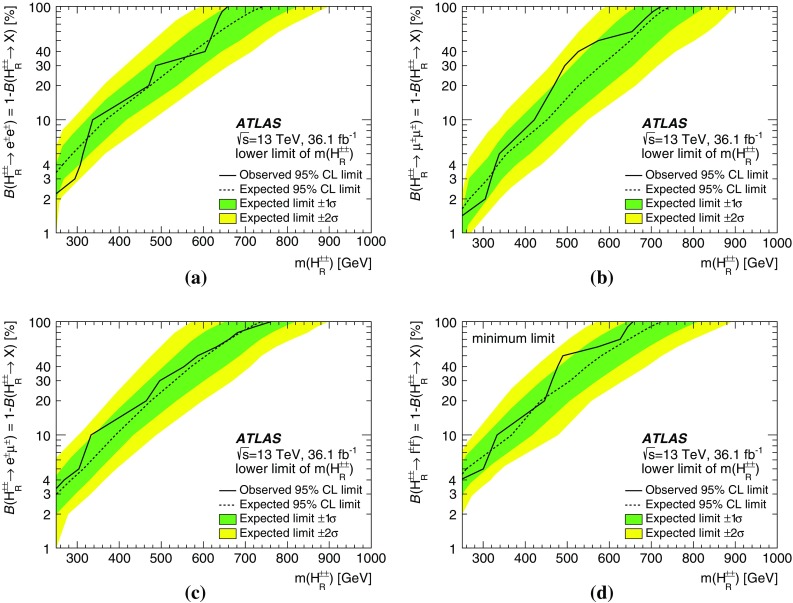
Lower limit on the $H_{R}^{\pm \pm }$ boson mass as a function of the branching ratio $B(H_{R}^{\pm \pm }\rightarrow \ell ^{\pm }\ell ^{\pm })$. Several cases are presented: **a** $H_{R}^{\pm \pm }$ decays only into electrons and “*X*”, **b** $H_{R}^{\pm \pm }$ decays only into muons and “*X*”, and **c** $H_{R}^{\pm \pm }$ decays only into electron–muon pairs and “*X*”, with “*X*” not entering any of the signal regions. Plot **d** shows the minimum observed and expected limit as a function of $B(H_{R}^{\pm \pm }\rightarrow \ell ^{\pm }\ell ^{\pm })$

The final result of the fit is a lower limit on the two-dimensional grid of the $H^{\pm \pm }$ boson mass for any combination of light lepton branching ratios that sum to a certain value. The fit was performed for values of $B(H^{\pm \pm }\rightarrow \ell ^{\pm }\ell ^{\pm })$ from $1\%$ to $5\%$ in $1\%$ intervals, and from $10\%$ to $100\%$ in $10\%$ intervals. Expected limits for $B(H^{\pm \pm }\rightarrow \ell ^{\pm }\ell ^{\pm })=100\%$ are presented in Fig. 11 for $H_{L}^{\pm \pm }$ and in Fig. 12 for $H_{R}^{\pm \pm }$. Results of the fit are presented in Figs. 13 and 14 for $H_L^{\pm \pm }$ and $H_R^{\pm \pm }$, respectively. Here, three specific decay scenarios to only $e^{\pm }e^{\pm }$, $\mu ^{\pm }\mu ^{\pm }$, and $e^{\pm }\mu ^{\pm }$, are considered and the minimum limit for each value of $B(H^{\pm \pm } \rightarrow \ell ^{\pm }\ell ^{\pm })$ is given. The minimum limit is obtained by taking, for each value of $B(H^{\pm \pm } \rightarrow \ell ^{\pm }\ell ^{\pm })$, the least stringent limit for any combination of branching ratios that sum to $B(H^{\pm \pm } \rightarrow \ell ^{\pm }\ell ^{\pm })$. The lower mass limits for these four cases are similar, which indicates that the analysis is almost equally sensitive to each decay channel.

The observed lower mass limits vary from 770 to 870 GeV for $H_{L}^{\pm \pm }$ with $B(H^{\pm \pm }\rightarrow \ell ^{\pm }\ell ^{\pm }) = 100\%$ and are above 450 GeV for $B(H^{\pm \pm }\rightarrow \ell ^{\pm }\ell ^{\pm }) \ge 10\%$. For $H_{R}^{\pm \pm }$ the lower mass limits vary from 660 to 760 GeV for $B(H^{\pm \pm }\rightarrow \ell ^{\pm }\ell ^{\pm })=100\%$ and are above 320 GeV for $B(H^{\pm \pm }\rightarrow \ell ^{\pm }\ell ^{\pm }) \ge 10\%$.

## Conclusion

The ATLAS detector at the Large Hadron Collider was used to search for doubly charged Higgs bosons in the same-charge dilepton invariant mass spectrum at high values, using $e^{\pm }e^{\pm }$, $e^{\pm }\mu ^{\pm }$ and $\mu ^{\pm }\mu ^{\pm }$ final states as well as final states with three or four leptons (electrons and/or muons). The search was performed with ${36.1} \text { fb}^{-1}$ of data from proton proton collisions at $\sqrt{s}=13\,\text {TeV}$, recorded during the 2015 and 2016 data-taking periods. No significant excess above the Standard Model prediction was found. As a result of the search, lower limits are set on the mass of doubly-charged Higgs bosons. These vary between 770 and 870 GeV for the $H_{L}^{\pm \pm }$ mass and for $B(H^{\pm \pm }\rightarrow \ell ^{\pm }\ell ^{\pm })=100\%$ and above 450 GeV for $B(H^{\pm \pm }\rightarrow \ell ^{\pm }\ell ^{\pm }) \ge 10\%$ for any combination of partial branching ratios. The observed lower limits on the $H_{R}^{\pm \pm }$ mass vary from 660 to 760 GeV for $B(H^{\pm \pm }\rightarrow \ell ^{\pm }\ell ^{\pm })=100\%$ and are above 320 GeV for $B(H^{\pm \pm }\rightarrow \ell ^{\pm }\ell ^{\pm }) \ge 10\%$. The observed limits are consistent with the expected limits. The lower limits on the $H_L^{\pm \pm }$ and $H_R^{\pm \pm }$ masses obtained in this search, under the assumption $B(H^{\pm \pm }\rightarrow \ell ^{\pm }\ell ^{\pm })=100\%$, are 300 GeV higher than those from the previous ATLAS analysis [27] and 450 GeV higher than those from the CMS analysis [28].

## Footnotes

1 ATLAS uses a right-handed coordinate system with its origin at the nominal interaction point (IP) in the centre of the detector and the *z*-axis along the beam pipe. The *x*-axis points from the IP to the centre of the LHC ring, and the *y*-axis points upwards. Cylindrical coordinates $(r,\phi )$ are used in the transverse plane, $\phi$ being the azimuthal angle around the *z*-axis. The pseudorapidity is defined in terms of the polar angle $\theta$ as $\eta = -\ln \tan (\theta /2)$. Angular distance is measured in units of $\Delta R \equiv \sqrt{(\Delta \eta )^{2} + (\Delta \phi )^{2}}$. Rapidity is defined as $y \equiv 0.5 \ln {[(E+p_{\text {z}})/(E-p_{\text {z}})]}$ where *E* denotes the energy and $p_{\text {z}}$ is the momentum component along the beam direction.

2 The probability of muon charge misidentification is negligible because muon tracks are measured both in the inner detector and in the muon spectrometer which provides a much larger lever arm for the curvature measurement.

3 The variable $p_{\text {T}} (\mathrm {\ell ^{\pm }\ell ^{\pm }})$ is the vector sum of the leptons’ transverse momenta.
